# Supplementary material for: A single-cell and spatial wheat root atlas with cross-species annotations delineates conserved tissue-specific marker genes and regulators
Source: Cell Rep. 2025 Feb 1;44(2):115240. doi: 10.1016/j.celrep.2025.115240 (PMC11860762; doi:10.1016/j.celrep.2025.115240)
Supplement: Document S1. Figures S1–S13 [file mmc1.pdf]

Cell Reports, Volume *44*

Supplemental information

A single-cell and spatial wheat root atlas with cross-species annotations delineates conserved tissue-specific marker genes and regulators

Yuji Ke, Vincent Pujol, Jasper Staut, Lotte Pollaris, Ruth Seurinck, Thomas Eekhout, Carolin Grones, Maite Saura-Sanchez, Michiel Van Bel, Marnik Vuylsteke, Andrea Ariani, Christophe Liseron-Monfils, Klaas Vandepoele, Yvan Saeys, and Bert De Rybel

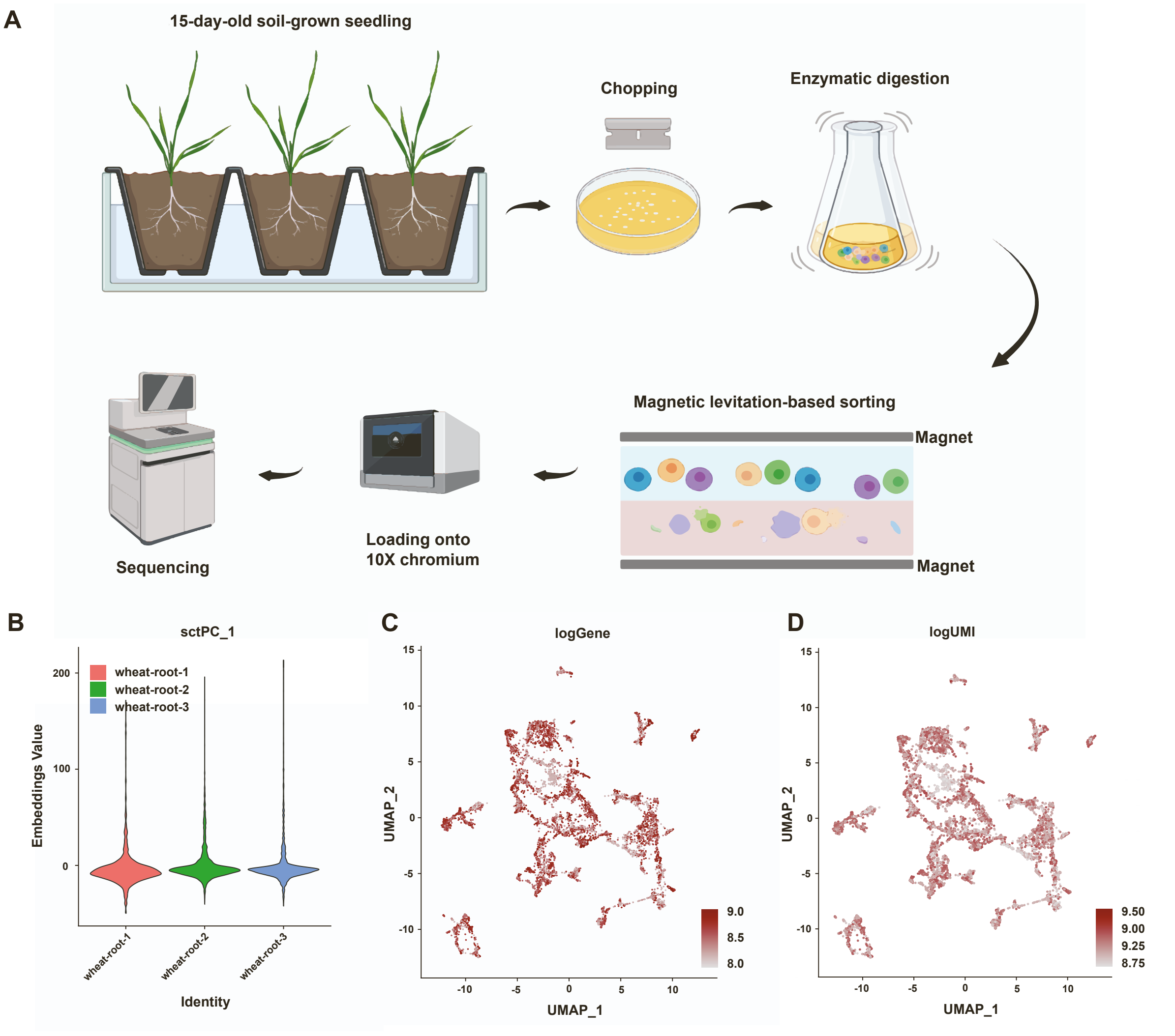

Figure S1. Experimental workflow and quality control of the wheat root apical meristem single-cell RNA-seq. (A) Overview of wheat scRNA-seq experimental workflow. Protoplasts were isolated from 5-mm root tips of 15-day soil-grown Chinese Spring wheat, cleaned using a magnetic sorter, loaded onto the 10x Genomics platform followed by high-throughput sequencing. (B) Violin plot showing the distribution of gene expression values for a specific principal component (sctPC_1) across three replicates. (C) Integrated UMAP showing Gene content distribution across cells. (D) Integrated UMAP showing UMI content distribution across cells.

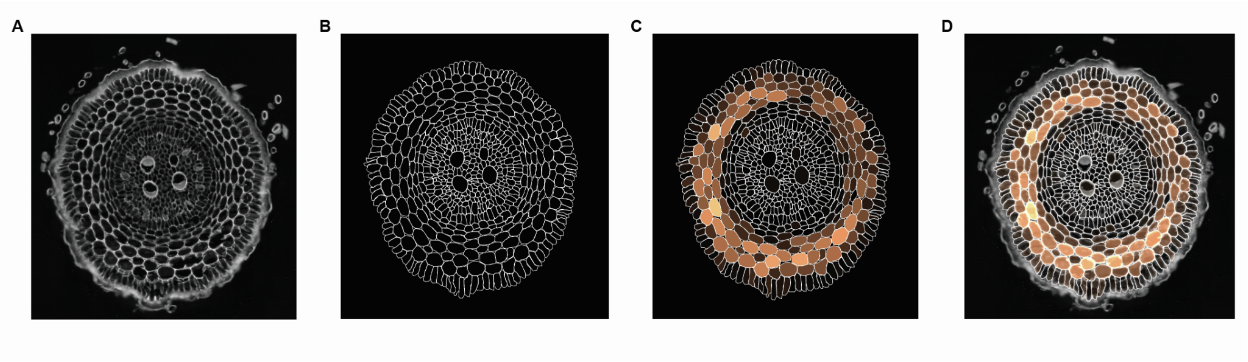

Figure S2. Stereo-Seq image processing. (A) Raw image of a root cross section stained with FB. (B) Manual segmentation of the root cross section. (C) Expression pattern of a cortex marker on segmented cells. (D) Overlay of expression pattern of a cortex marker, segmented cells, and raw image of the root section.

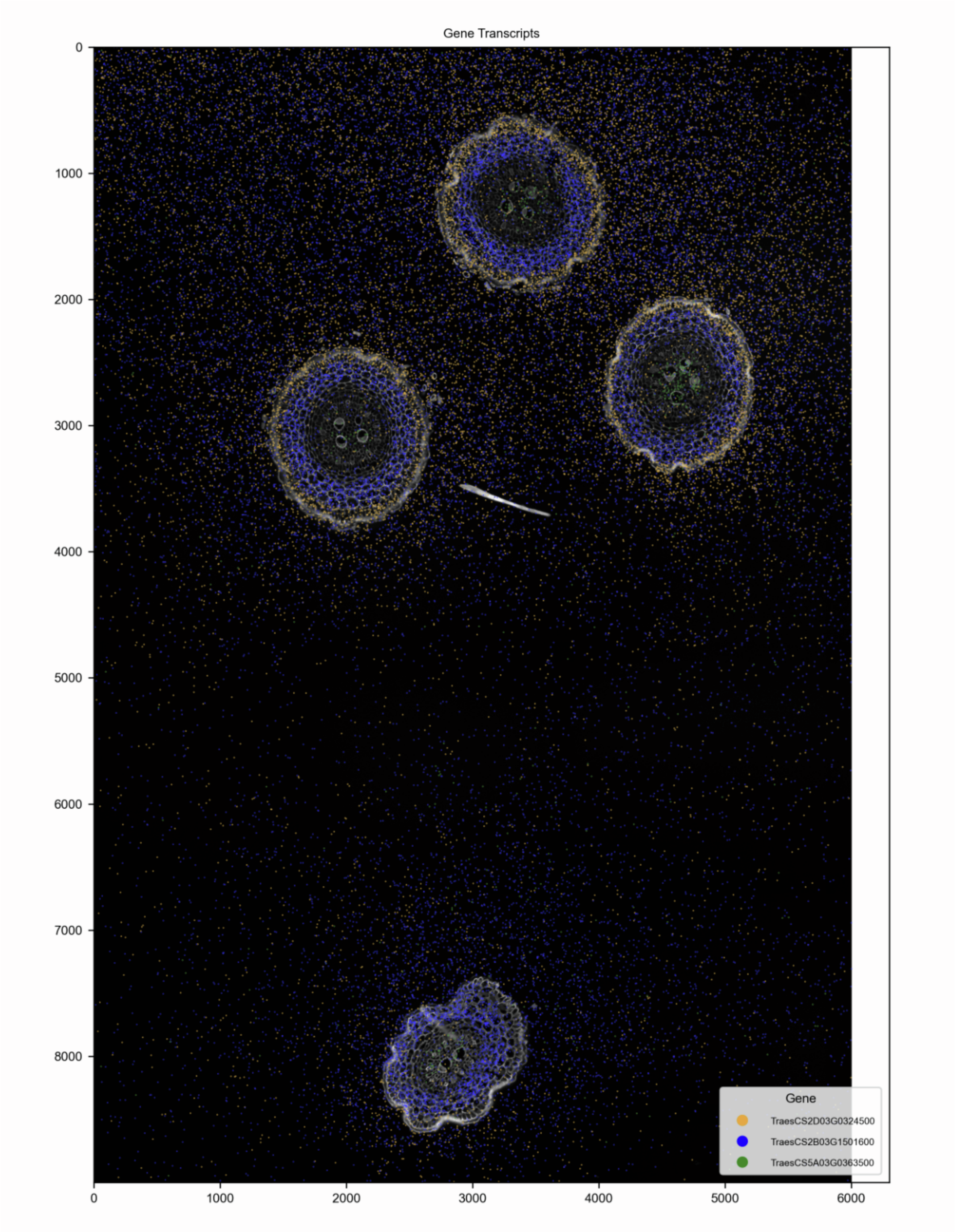

Figure S3. Expression pattern of an epidermis, cortex, and provascular cells marker gene on unsegmented Stereo-seq section image. Dots outside the tissue section indicate transcript diffusion.

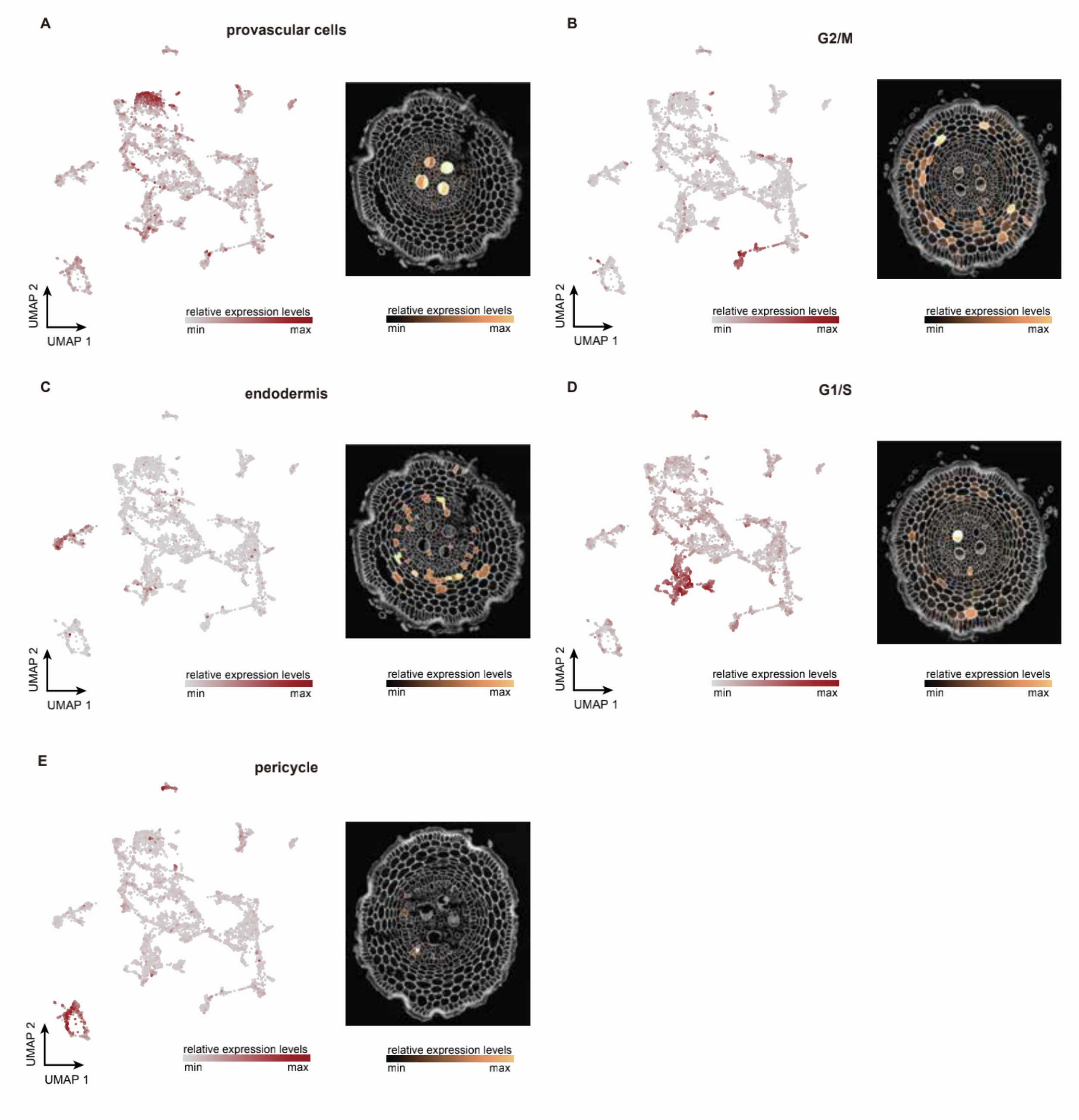

Figure S4. scRNA-seq derived marker gene expression patterns in Stereo-seq root sections. (**A-D**) UMAP feature plot and Stereo-seq visualization of marker genes from provascular cells (A), G2/M (B), endodermis (C), G1/S (D), pericycle (E).

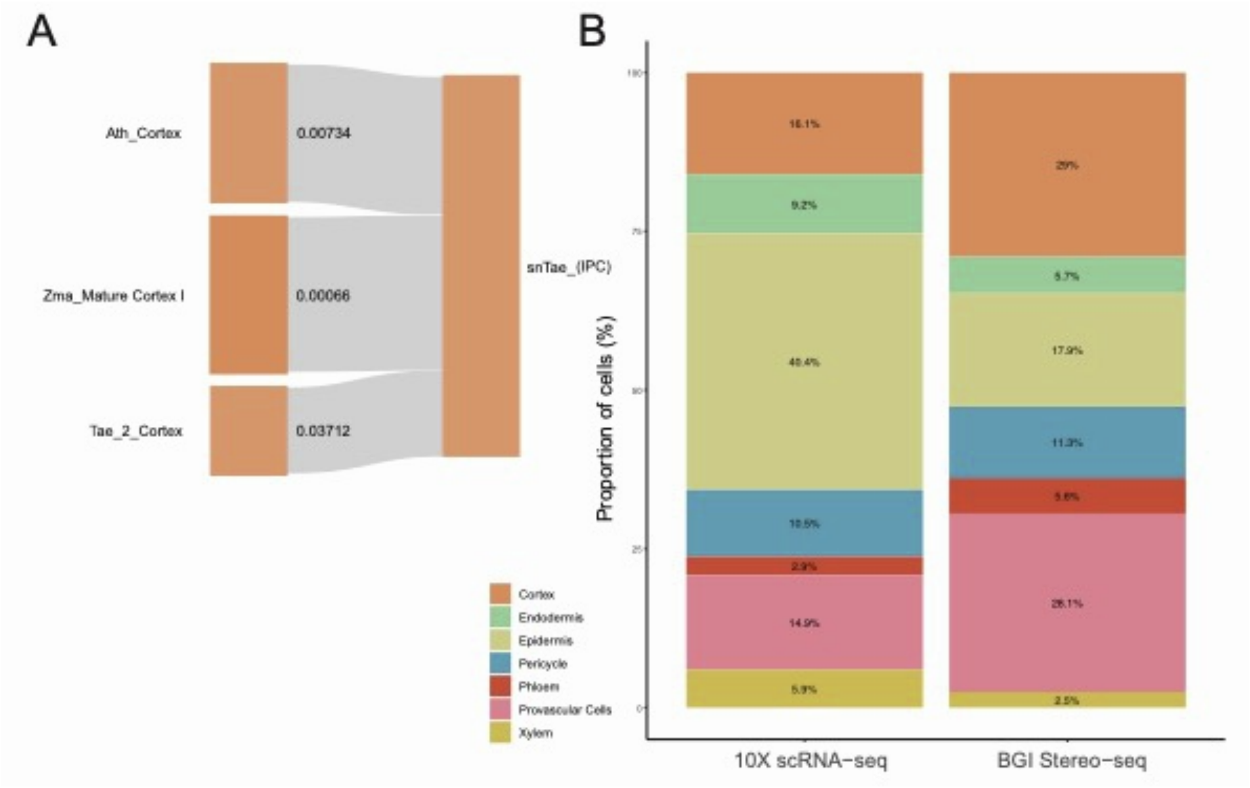

Figure S5. Resolved cell type annotation and cell type composition of single cell (10X genomics) and spatial transcriptomics (STOmics Stereo-seq) dataset. (A) Sankey plot showing resolved annotations transferred from Arabidopsis (*Ath*), maize (*Zma*), wheat (*Tae*) to snTae_Immature Pericycle Cells (IPC) and corresponding q-value. (**B**) Proportion of major cell types in the scRNA-seq and spatial transcriptomics dataset for comparison.

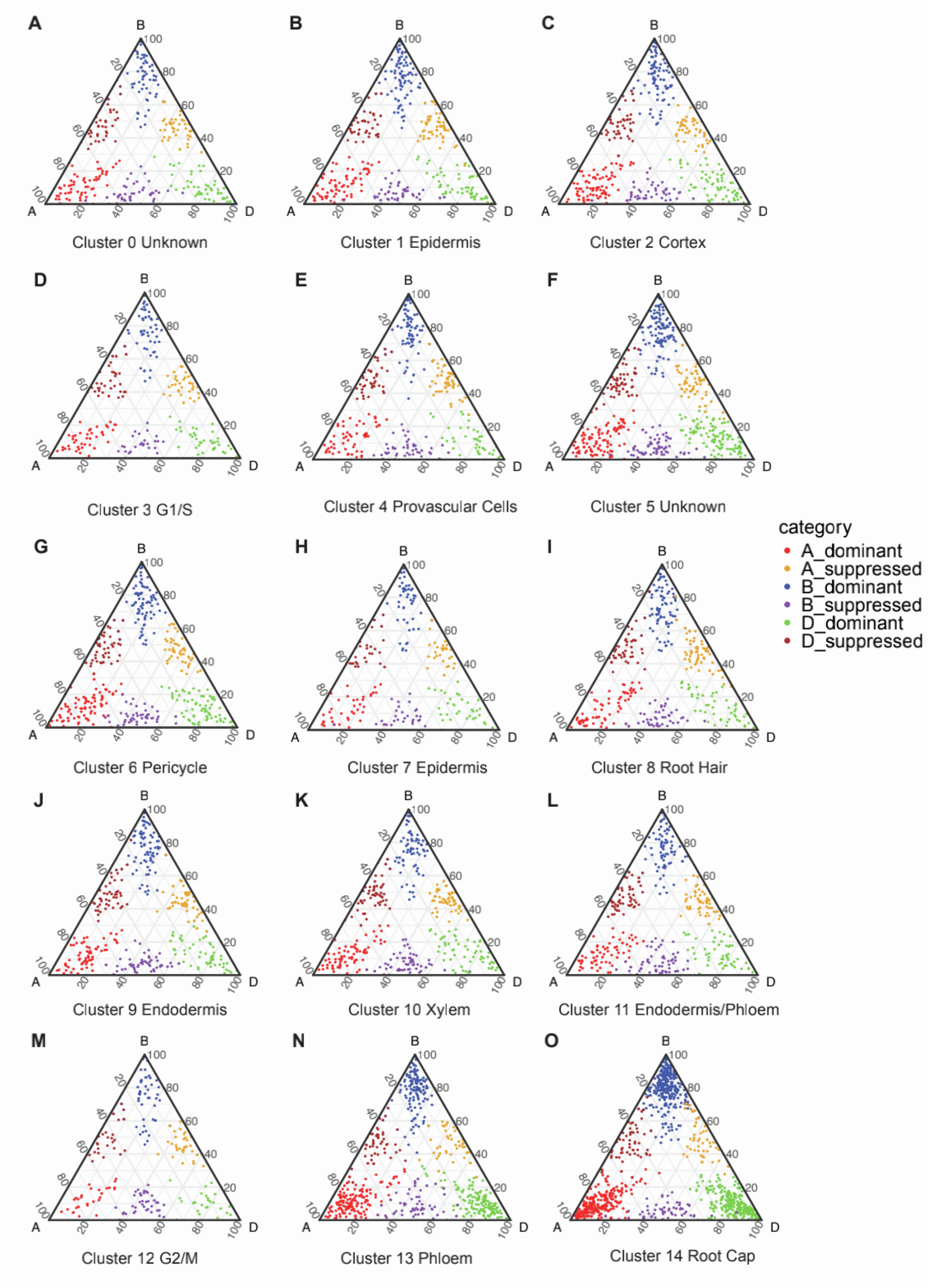

Figure S6. Ternary plots showing genome asymmetry distribution across all clusters.

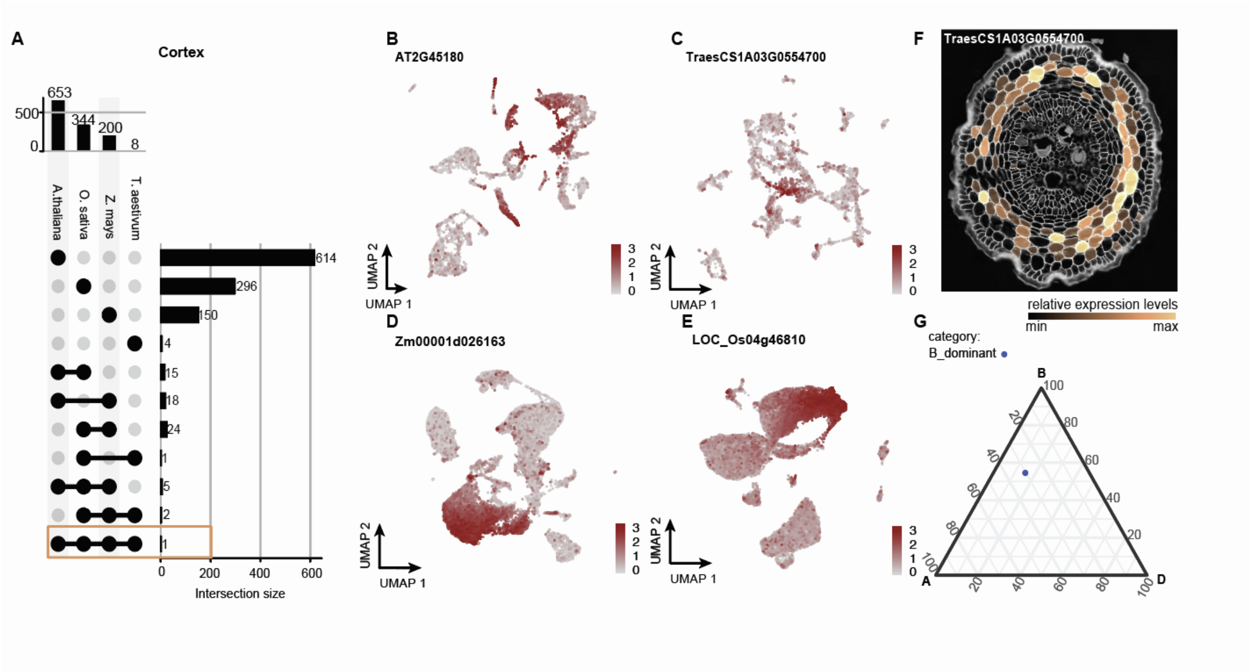

Figure S7. Tissue-specific markers conserved across Arabidopsis, wheat, rice, and maize. (**A**) UpSet plot showing the intersections of cortex markers across Arabidopsis, wheat, rice, and maize. (**B-E**) Feature plots of a cortex specific marker across species. (**F-G**) Spatial expression in Stereo-seq data (F) and ternary plot showing genome asymmetry information (G) of the same cortex specific marker in the wheat root meristem.

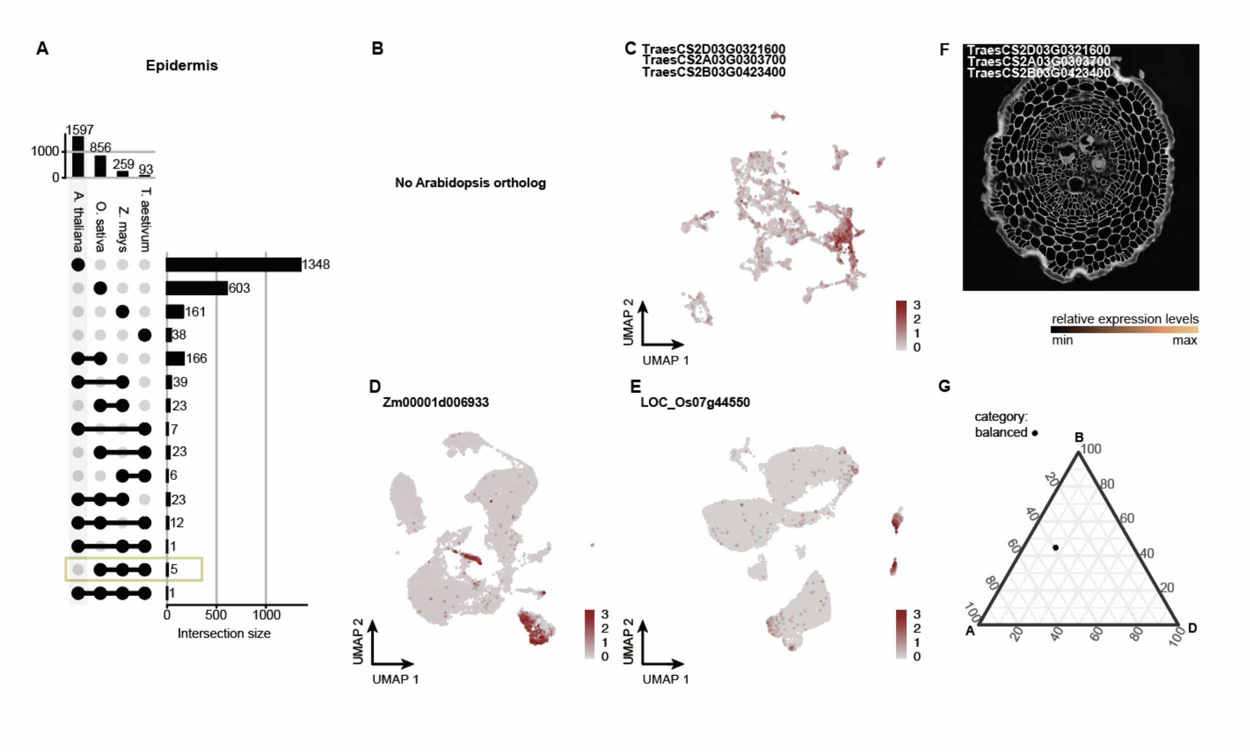

Figure S8. Tissue-specific markers unique to monocot clade (wheat, rice, and maize). (**A**) UpSet plot showing the intersections of epidermis markers across Arabidopsis, wheat, rice, and maize. (**B-E**) Feature plots of an epidermis specific marker across species. (**F-G**) Spatial expression in Stereo-seq data (F) and ternary plot showing genome asymmetry information (G) of the same epidermis specific marker in the wheat root meristem.

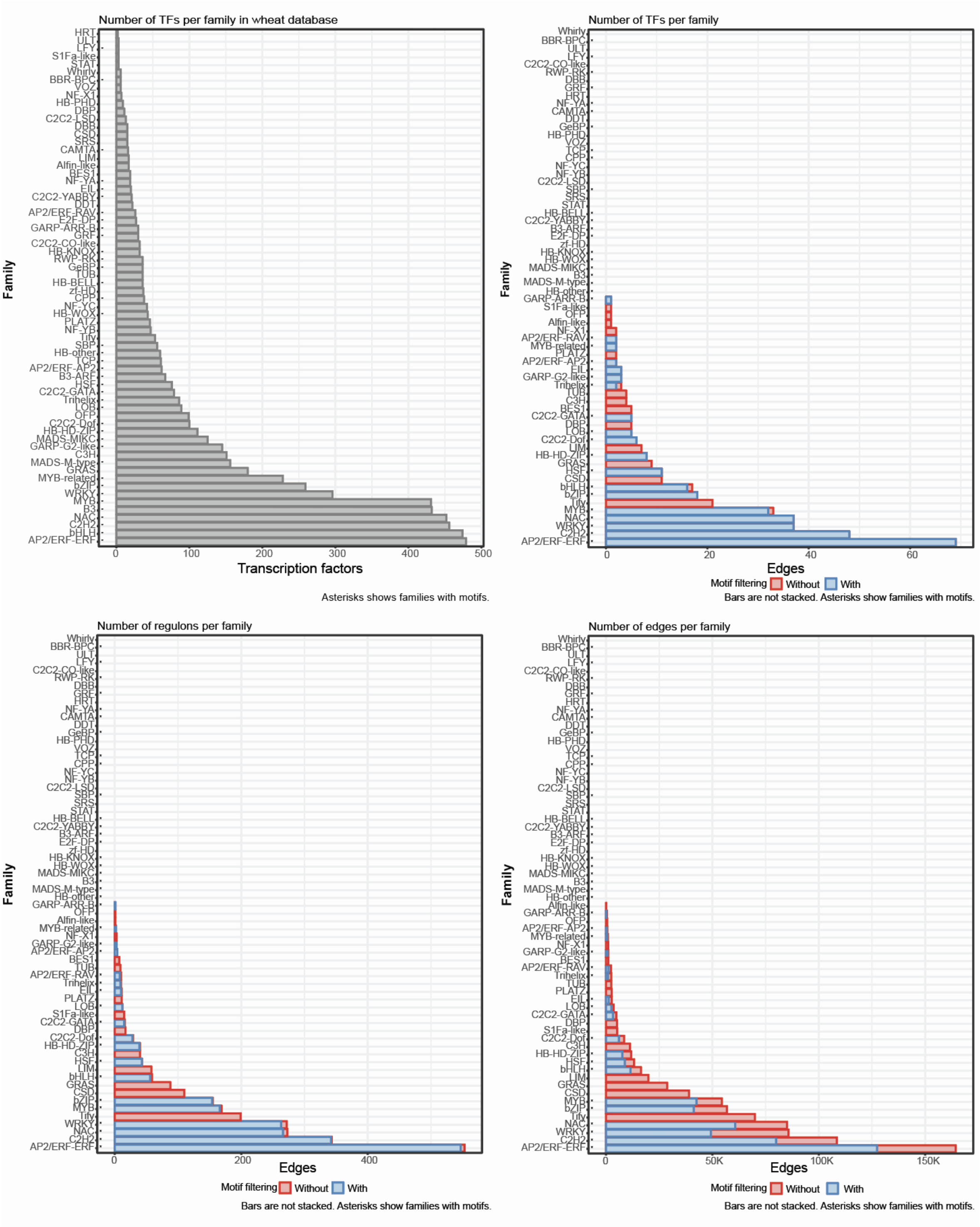

Figure S9. Impact of motif enrichment analysis on MINI-EX results for wheat. (A) Distribution of TFs across TF families within the MINI-EX wheat database. (B-C-D) Comparison of TFs, regulons and edges across TF families in MINI-EX results with or without motif enrichment analysis. Some families are entirely excluded from the GRN as they lack associated motifs.

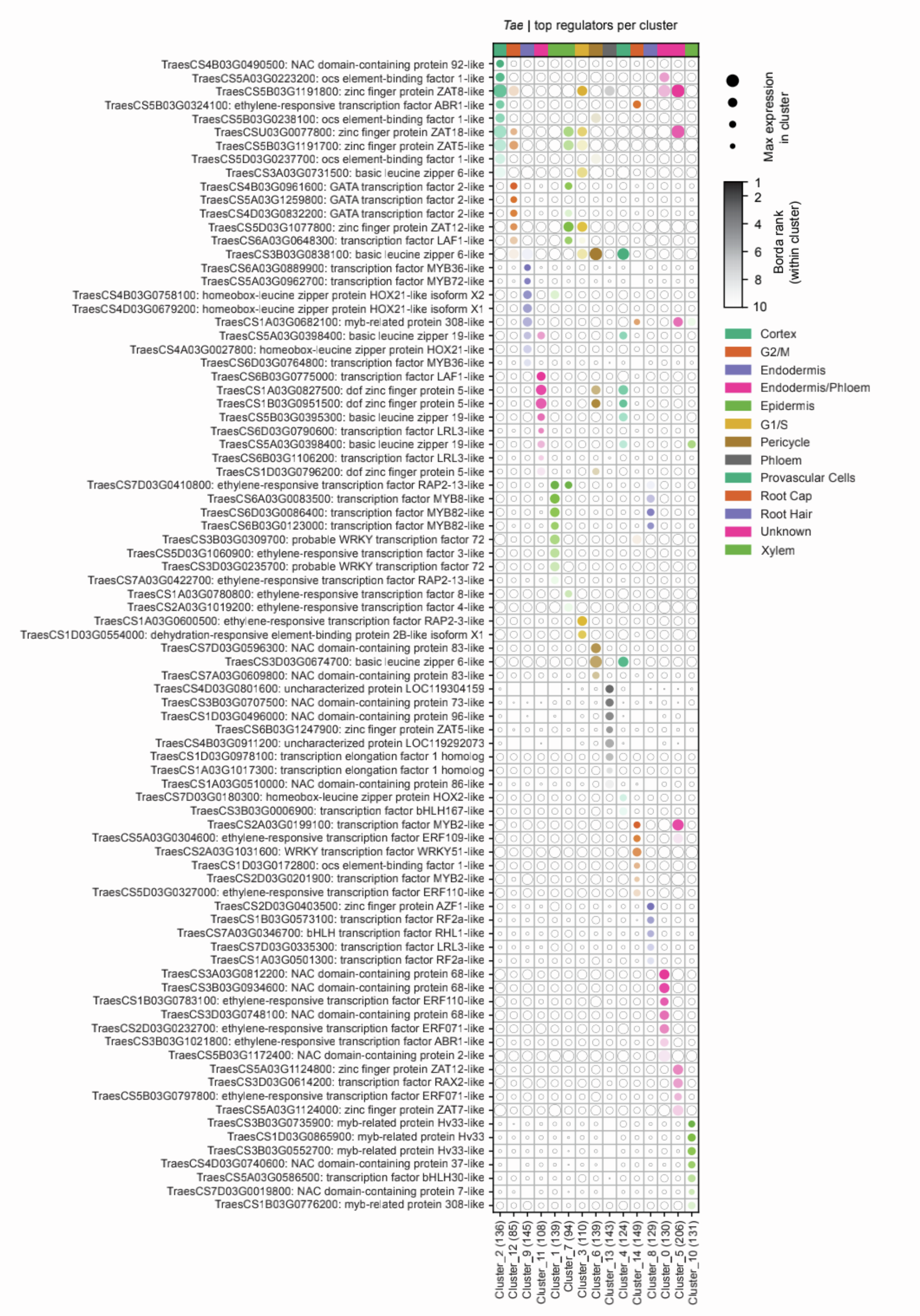

Figure S10. Top regulons for each cluster for wheat (*Tae*).

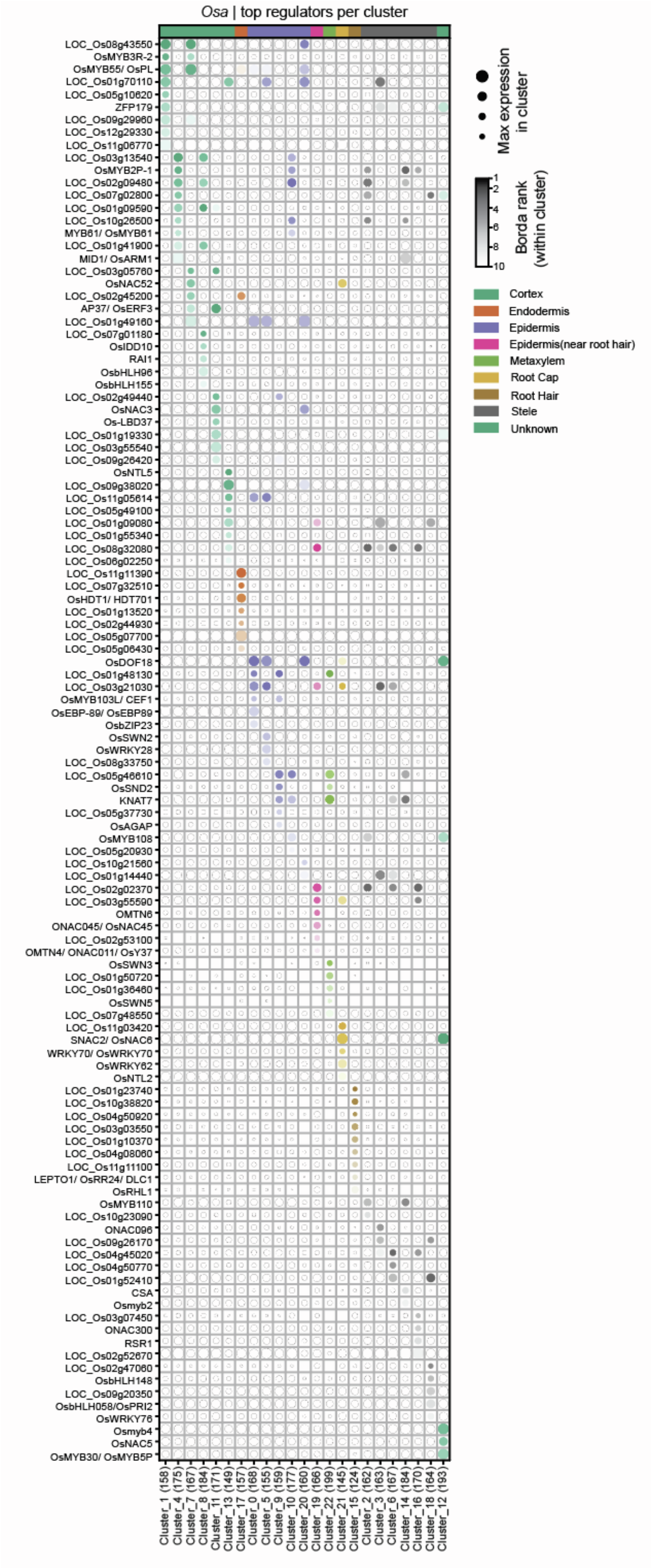

Figure S11. Top regulons for each cluster for rice (*Osa*).

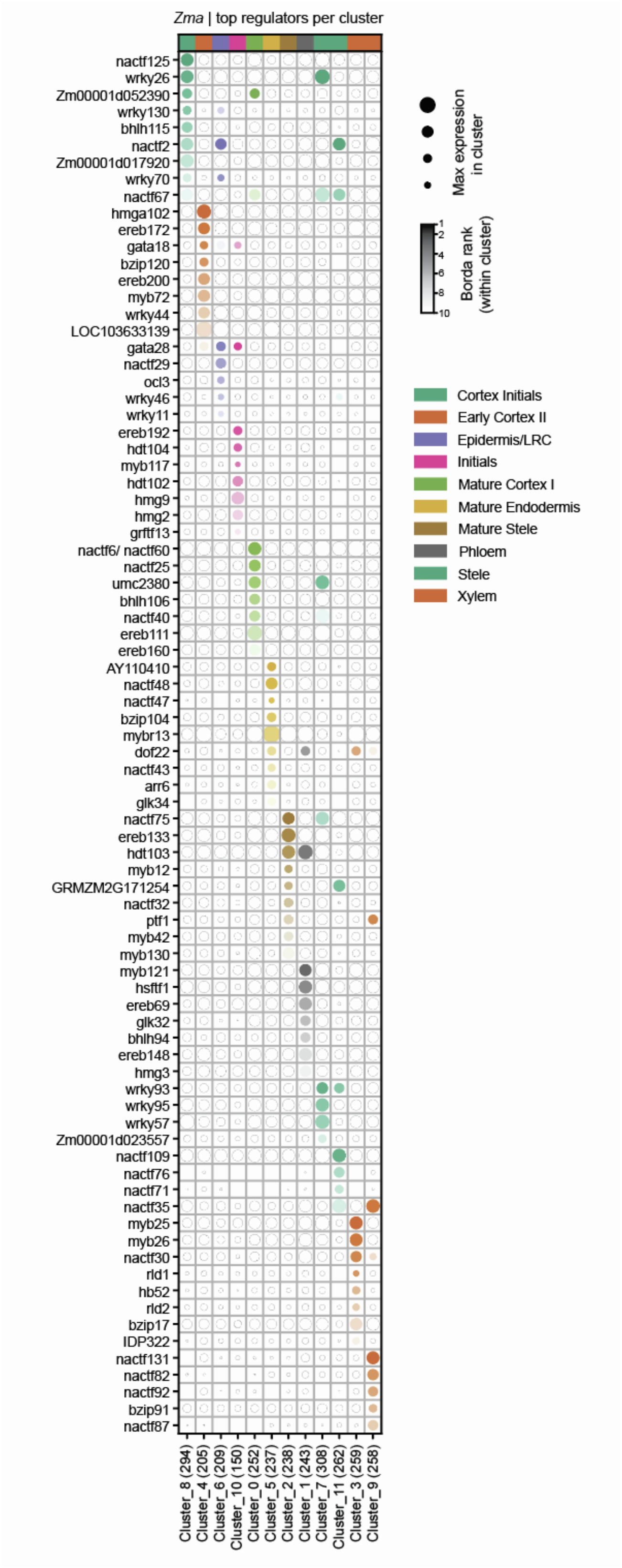

Figure S12. Top regulons for each cluster for maize (*Zma*).

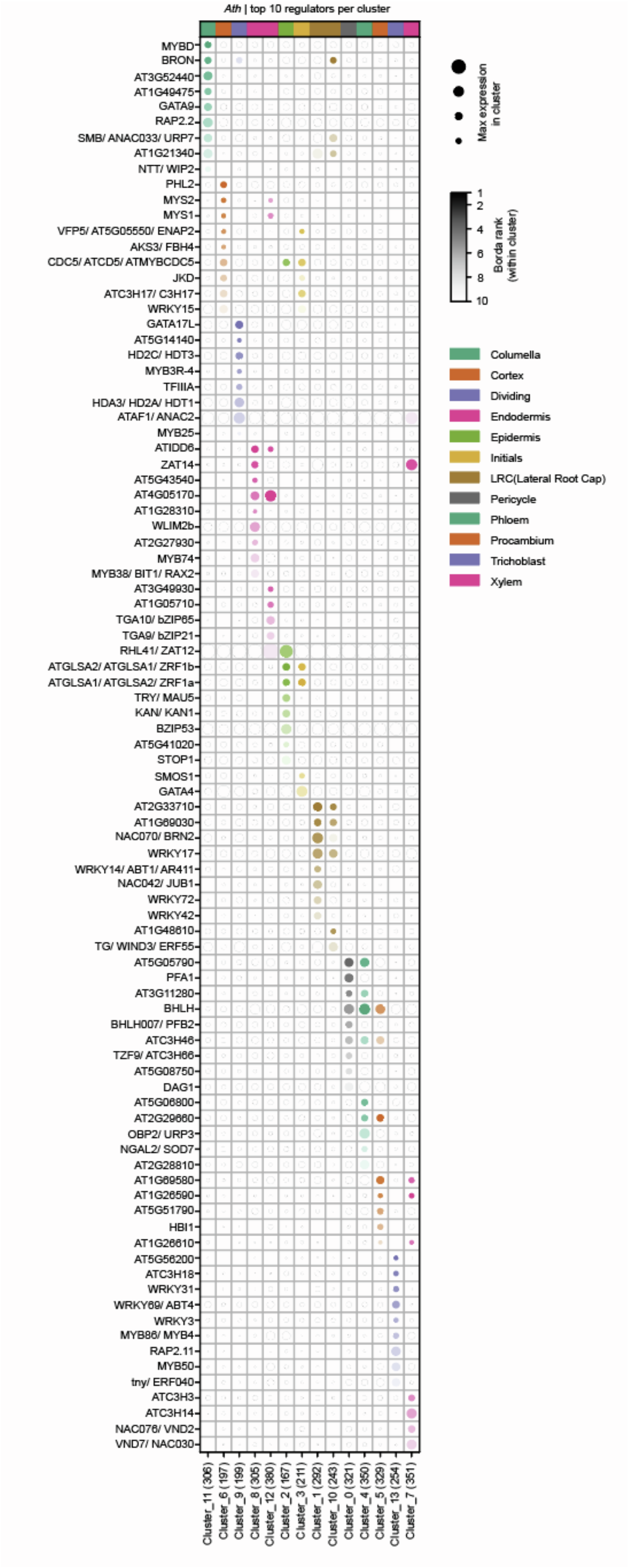

Figure S13. Top regulons for each cluster for Arabidopsis (*Ath*).
