# Supplementary material for: A single-cell and spatial wheat root atlas with cross-species annotations delineates conserved tissue-specific marker genes and regulators
Source: Cell Rep. 2025 Feb 1;44(2):115240. doi: 10.1016/j.celrep.2025.115240 (PMC11860762; doi:10.1016/j.celrep.2025.115240)
Supplement: Document S2. Article plus supplemental information [file mmc14.pdf]

Cell Reports

Article

A single-cell and spatial wheat root atlas with cross-species annotations delineates conserved tissue-specific marker genes and regulators

Graphical abstract

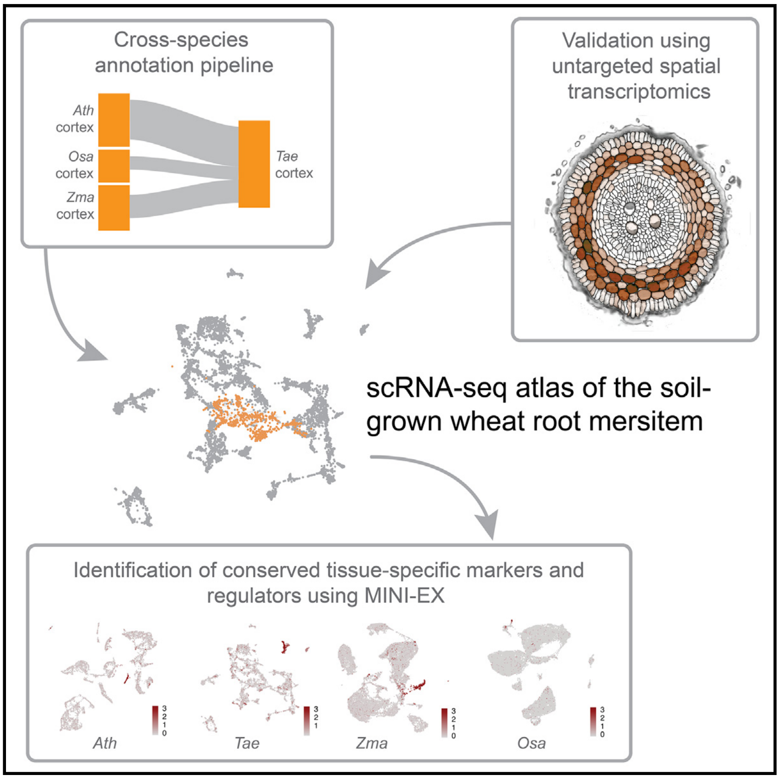

Highlights

- A single-cell soil-grown wheat root atlas validated by untargeted spatial transcriptomics
- An orthology-based approach provides reliable cluster annotations in wheat
- Cross-species analysis reveals evolutionarily conserved cell type-specific marker genes
- Single-cell GRN analysis identifies known and uncharacterized developmental regulators

Authors

Yuji Ke, Vincent Pujol, Jasper Staut, ..., Klaas Vandepoele, Yvan Saeys, Bert De Rybel

Correspondence

klaas.vandepoele@psb.vib-ugent.be (K.V.),
yvan.saeys@ugent.be (Y.S.),
bert.derybel@psb.vib-ugent.be (B.D.R.)

In brief

Accurate scRNA-sequencing cluster annotation in less-studied plant species remains a major challenge due to the lack of validated marker genes. Ke et al. provide an orthology-based annotation pipeline and apply it to a soil-grown wheat root apical meristem dataset, which is validated using untargeted spatial transcriptomics.

Ke et al., 2025, Cell Reports *44*, 115240
February 25, 2025 © 2025 The Author(s). Published by Elsevier Inc.
https://doi.org/10.1016/j.celrep.2025.115240

CellPress

Cell Reports

CellPress
OPEN ACCESS

Article

A single-cell and spatial wheat root atlas with cross-species annotations delineates conserved tissue-specific marker genes and regulators

Yuji Ke,[1,2,10] Vincent Pujol,[3,4,10] Jasper Staut,[1,2,10] Lotte Pollaris,[3,4] Ruth Seurinck,[3,4] Thomas Eekhout,[1,2,5] Carolin Grones,[1,2,8] Maite Saura-Sanchez,[1,2] Michiel Van Bel,[1,2] Marnik Vuylsteke,[6] Andrea Ariani,[7] Christophe Liseron-Monfils,[7,9] Klaas Vandepoele,[1,2,*] Yvan Saeys,[3,4,*] and Bert De Rybel[1,2,11,*]

[1]Department of Plant Biotechnology and Bioinformatics, Ghent University, Ghent, Belgium
[2]VIB Center for Plant Systems Biology, Ghent, Belgium
[3]Department of Applied Mathematics, Computer Science and Statistics, Ghent University, Ghent, Belgium
[4]VIB Center for Inflammation Research, Ghent, BE, Belgium
[5]VIB Single Cell Core, VIB, Ghent/Leuven, Belgium
[6]Gnomixx, Melle, Belgium
[7]BASF Belgium Coordination Center CommV, Innovation Center Gent, Technologiepark-Zwijnaarde 101, 9052 Ghent, Belgium
[8]Present address: Laboratory of Cell and Developmental Biology, Cluster of Plant Developmental Biology, Department of Plant Sciences, Wageningen University, Droevendaalsesteeg 1, 6708 PB Wageningen, the Netherlands
[9]Present address: National Research Council Canada, 110 Gymnasium Place, Saskatoon, SK S7N 0W9, Canada
[10]These authors contributed equally
[11]Lead contact
*Correspondence: klaas.vandepoele@psb.vib-ugent.be (K.V.), yvan.saeys@ugent.be (Y.S.), bert.derybel@psb.vib-ugent.be (B.D.R.)
https://doi.org/10.1016/j.celrep.2025.115240

SUMMARY

Despite the broad use of single-cell/nucleus RNA sequencing in plant research, accurate cluster annotation in less-studied plant species remains a major challenge due to the lack of validated marker genes. Here, we generated a single-cell RNA sequencing atlas of soil-grown wheat roots and annotated cluster identities by transferring annotations from publicly available datasets in wheat, rice, maize, and *Arabidopsis*. The predictions from our orthology-based annotation approach were next validated using untargeted spatial transcriptomics. These results allowed us to predict evolutionarily conserved tissue-specific markers and generate cell type-specific gene regulatory networks for root tissues of wheat and the other species used in our analysis. In summary, we generated a single-cell and spatial transcriptomics resource for wheat root apical meristems, including numerous known and uncharacterized cell type-specific marker genes and developmental regulators. These data and analyses will facilitate future cell type annotation in non-model plant species.

INTRODUCTION

Since the first set of studies describing the application of single-cell transcriptomics in *Arabidopsis thaliana* root meristems,[1–7] single-cell RNA sequencing (scRNA-seq) and single-nucleus RNA sequencing (snRNA-seq) are rapidly being integrated in plant research.[8–14] The use of this technology is no longer limited to *Arabidopsis* roots, as multiple tissues, organs, and plant species are being profiled, with developing ears in maize,[15] rice pistils,[16] soybean nodules,[17] and pitaya pericarp[18] as a few examples. Given their importance for food security, applying sc/snRNA-seq to monocot crop species is of specific interest.[19] However, despite its importance for global food security, available single-cell/nucleus transcriptomics resources for wheat remain scarce, with only one available single-nucleus dataset for roots[20] and one single-cell dataset for the coleoptile,[21] compared to multiple studies across various tissue types in other economically important crops, such as maize[15,22–31] and rice.[16,32–38]

For less-intensely studied species like wheat, reliable cell type annotation is a major challenge in sc/snRNA-seq analyses due to the scarcity of available markers in addition to the loss of spatial information during protoplast generation or nucleus isolation. To circumvent this problem, orthology to well-characterized and/or validated cell type and cell state markers in other species is utilized. Often, this involves homology to markers in *Arabidopsis*, for which a wealth of information is available through validated transcriptional reporter lines and high-throughput transcriptome profiling on isolated cell types and cell states, specifically in root tissues.[39,40] However, relying solely on marker genes from *Arabidopsis* for cell type annotation has not always proven to be successful,[32] likely due to anatomic specialization of cell types and functional divergence among genes underlying similar cell types in *Arabidopsis* and other species. Furthermore, validation through mRNA *in situ* hybridization or mRNA fluorescence *in situ* hybridization[15,28,32] is labor intensive, low throughput, and highly dependent upon probe design and signal intensity.[41] All

© 2025 The Author(s). Published by Elsevier Inc.
This is an open access article under the CC BY license (http://creativecommons.org/licenses/by/4.0/).

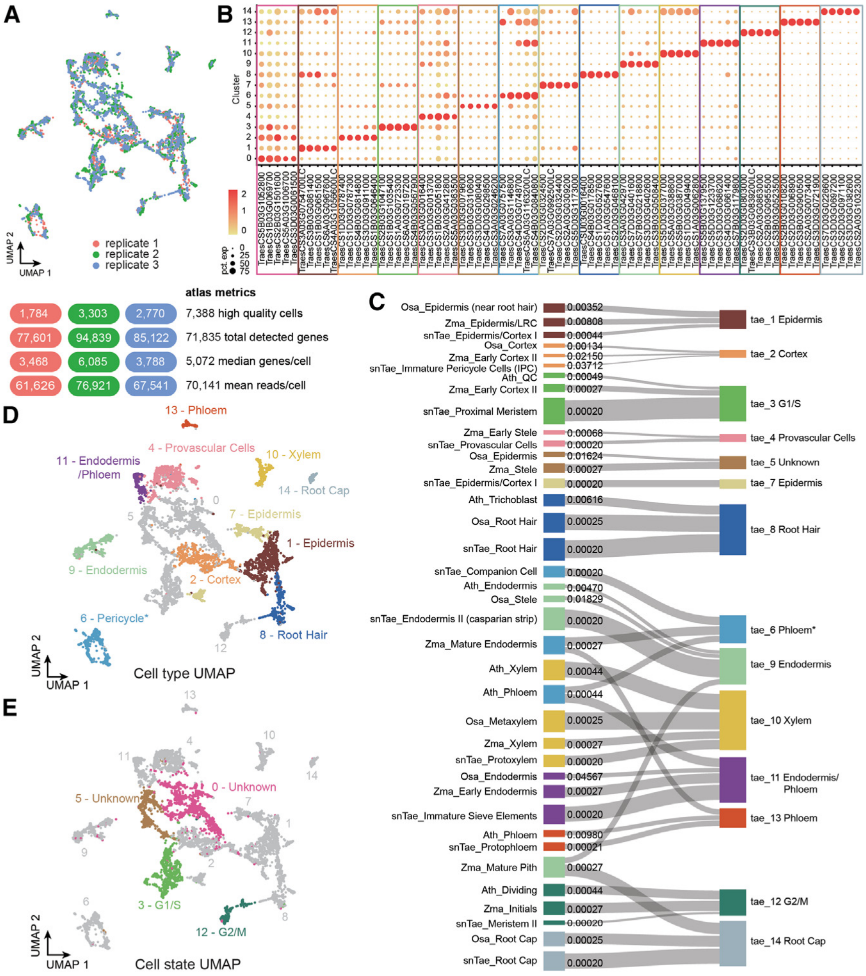

Figure 1. Single-cell RNA-seq and cluster annotation of wheat root tips

(A) UMAP visualization of the three replicates in our scRNA-seq experiment and corresponding atlas metrics.

(B) Expression of cell type markers across each cluster. Dot diameter, proportion of cluster cells in a cluster expressing a given gene; color, mean expression across cells in that cluster.

(C) Sankey plot showing annotations transferred from *Arabidopsis* (*Ath*), rice (*Osa*), maize (*Zma*), and single-nuclei wheat (sn*Tae*) to our wheat atlas (*Tae*) and corresponding q value.

(D and E) Annotated UMAPs with cell type (D) and cell state (E) annotations. Please note that cluster 6 was manually annotated as pericycle based on evidence from STOmics Stereo-seq data and known pericycle marker genes and was therefore marked with an asterisk.

of these considerations limit the high-throughput use of cell type markers for cluster annotation and overall sc/snRNA-seq data validation in crop species like wheat. Instead, validation using targeted[28,30,42] and untargeted spatial transcriptomics technologies[17,18,38,43–47] is gaining traction and demonstrating efficacy.

RESULTS

Generation of a single-cell soil-grown wheat root apical meristem atlas

To characterize cell type gene expression in a plant species with a complex polyploid genome and expand the available resources for the research community, we generated a single-cell transcriptome atlas for wheat root apical meristems under realistic growth conditions. We applied droplet-based scRNA-seq on dissected root apical meristems (bottom 0.5 cm of the root meristem) harvested from 15-day-old soil-grown allohexaploid bread wheat seedlings (*Triticum aestivum* cultivar Chinese Spring, 2N = 6× = 42, AABBDD) in three replicates (Figures S1A and 1A). The soil-grown root tissues were enzymatically digested into protoplasts using an optimized protocol (see STAR Methods for details); protoplasts were enriched, and debris was removed in a gentle way using the LeviCell platform (Figure S1A). After library preparation, sequencing and quality control (Figures S1B–S1D; see STAR Methods for details), this resulted in a soil-grown wheat root meristem atlas containing 7,388 high-quality cells with a total of 71,835 expressed genes (see Table S1 for detailed metadata). We visualized the data with dimensionality reduction using uniform manifold approximation and projection (UMAP) and grouped cells into 15 transcriptionally distinct clusters. For each of these clusters, we predicted a set of highly cluster-specific genes (Figure 1B), among which we found genes orthologous to known marker genes reported in other studies (Table S2), which can serve as marker genes for future use in wheat root tissues.

Predicting cluster annotations through cross-species orthology

In most plant species and organs, there are very few to no experimentally validated marker genes that could be used to reliably annotate cell identities to clusters obtained from single-cell approaches. Even when marker genes exist, it is questionable whether annotating entire cluster identities based on one or two marker genes is the best approach.[14] Alternative methods make use of a few known validated orthologous markers from the well-annotated model eudicot species *Arabidopsis thaliana* to annotate less-intensely studied species like *Medicago truncatula*, poplar, and tomato.[48–50] This method is only as good as the available marker genes and depends highly on the evolutionary distance between *Arabidopsis* and the species to be queried. In another work, a set of single-copy orthologs from maize, sorghum, and *Setaria* was first used to co-cluster the expression profiles of cells/nuclei in the three species, followed by cluster annotation using known maize cell type markers.[28] However, this approach does not translate well to species with a more complex genome, such as allohexaploid wheat, containing

homoeologous genes that can undergo expression divergence.[51] To circumvent these limitations, we implemented a statistical framework to map existing cluster annotations to new, unannotated datasets using cross-species orthology. While some individual orthologs can exhibit diverging cell type specificity, at the group level they generally tend to display similar cell type specificities, allowing them to be used to transfer cell type information across species. To manage the variability in cell type specificity among orthologs, we employed an enrichment approach to detect overrepresentation of tissue-specific markers. By calculating the enrichment of orthologous groups of genes, we can avoid the limitations of single-copy orthologs. The resulting increase in the number of marker genes allows us to more reliably transfer cluster annotations to the allopolyploid wheat dataset (see below and Table S3).

To annotate our clusters, we used scRNA-seq information from other major cereal crop species for which more molecular information and validated cluster identities are available, including maize (*Zma*),[24] rice (*Osa*),[32] and a previously published wheat single-nucleus RNA-seq study (sn*Tae*).[20] Although evolutionarily more distant from these monocot species, we also included *Arabidopsis* (*Ath*),[7] as this is still the best-validated model species, especially when it comes to the root apical meristem. Our approach for transferring cluster annotations to other species starts by computationally inferring cluster-specific marker genes or differentially expressed genes (DEGs). We inferred these for our soil-grown wheat atlas (STAR Methods) and retrieved DEGs as provided by publicly available *Arabidopsis*, maize, rice, and single-nucleus wheat datasets. These DEGs form the basis of our annotation approach. To compare them across species, we inferred orthologous groups using a carefully selected set of species (see STAR Methods for details). To identify similar clusters in different species, our approach converts DEGs into their corresponding orthologous group and then generates a background distribution of these groups. This background consists of orthologous groups that are randomly drawn from all orthologous groups that are observed as a marker in any cluster. By comparing the real overlap in orthologous groups between two clusters with the overlap with the background sets, a fold enrichment change and corresponding *p* value can be calculated. This statistical approach indicates how likely it is that the overlap in orthologous groups between the two clusters is observed only by chance. This procedure is applied to all pairwise combinations of clusters between species, and *p* values are corrected for multiple testing (q value) (Table S3; Figure 1C). In case of a comparison between the same species, the DEGs instead of the orthologous groups are compared analogously. Annotations from the publicly available datasets for *Arabidopsis*, rice, maize, and wheat were transferred to the clusters of our newly generated dataset using the best hit (smallest q value, largest fold enrichment to break ties) in each public dataset. Since available datasets annotate clusters at different levels of resolution, we grouped all cluster identities into main tissue types: epidermis, cortex, endodermis, pericycle, xylem, phloem, and root cap. To avoid confusion between clusters with clear annotation at the anatomical level and clusters more clearly defined by cellular processes/states, we separated cell states and cell types in UMAP representations.[14] This unbiased approach predicted cluster annotations of all major cell types, including epidermis, cortex, endodermis, xylem, phloem, and root cap, for the newly generated wheat scRNA-seq dataset (Figure 1D). The fact that our approach is able to annotate the major cell types despite the lower sequencing depth due to the large number of expressed genes in the polyploid wheat genome further supports the feasibility of our method. Note that the pericycle was not annotated, as this cell identity was not annotated in several of the datasets used for our analysis. Additionally, G2/M and G1/S phases were also annotated as distinct cell states (Figure 1E). Our approach allowed us to annotate clusters using more marker genes than through a manual or single-copy ortholog approach (Table S3). Indeed, the single-copy approach retrieves only 33 genes among wheat, rice, maize, and *Arabidopsis*, none of which are also wheat DEGs, rendering this approach not usable in our case. As one example of using our statistical approach, we annotated cluster *Tae_4* as provascular cells using 6 genes from the wheat snRNA-seq dataset. One of these genes refers to *AtWAT1*, which is the only validated marker previously used to manually annotate the provascular cluster in the wheat snRNA-seq dataset.[20] Therefore, our approach not only revealed a known provascular marker gene but simultaneously revealed more candidate marker genes. Therefore, we provided a list of overlapping genes/matches used for all other cross-species annotations (Table S3). Overall, our cross-species orthology approach managed to predict all major cell type/cell states in our newly generated wheat root apical meristem atlas and to simultaneously provide more candidate marker genes that can be used to infer cell identities in other species.

Validation of the cluster annotations using spatial transcriptomics

To experimentally validate the predicted annotations of our soil-grown wheat root meristem atlas obtained from the orthology-based mapping approach, we next optimized and implemented an untargeted spatial transcriptomics (ST) technology called STOmics Stereo-seq[44,52] on the same samples as collected for scRNA-seq experiment (see STAR Methods for experimental and analysis details). After processing the images (Figure S2), we examined the top DEGs of each cluster identified through scRNA-seq (Table S4) across all available segmented sections. Although we noticed transcript diffusion outside the tissue sections (Figure S3), plotting the top DEGs for the predicted epidermis (cluster 7), cortex (cluster 2), xylem (cluster 10), phloem (cluster 13), root cap (cluster 14), G1/S (cluster 3), G2/M (cluster 12), provascular cells (cluster 4), and endodermis (cluster 9) clusters showed the expected expression patterns (Table S5; Figures 2B–2F and S4A–S4D; STAR Methods). We next determined the overlap in cluster annotation between the untargeted ST data showing gene expression *in situ* on tissue sections and the predictions from our orthology-based approach on the scRNA-seq data. On average, 62.8% (113 of 180) of the top 20 DEGs of each scRNA-seq cluster annotated using our orthology-based approach overlapped in the spatial expression patterns, validating these genes as cell type/state-specific marker genes (Table S5). As a general observation, the overlap was very high for tissues with many cells on the tissue sections of the untargeted ST and poor for tissue types with few cells

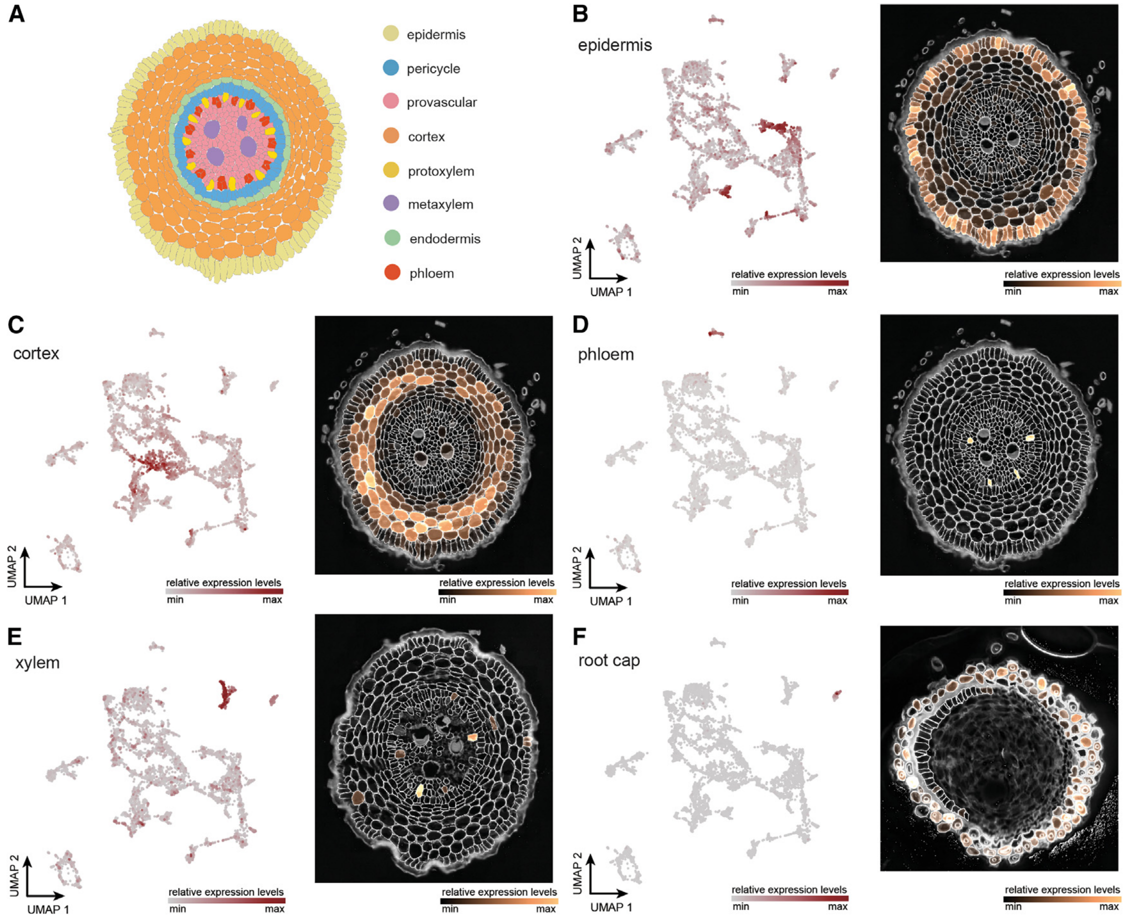

Figure 2. scRNA-seq-derived marker gene expression patterns in STOmics Stereo-seq root sections

(A) A cross-section of wheat root apical meristem with major cell types annotated.

(B–F) UMAP feature plot and STOmics Stereo-seq visualization of marker genes from epidermis (B), cortex (C), phloem (D), xylem (E), and root cap (F).

on the sections. Thus, although the majority of genes in the top 20 DEGs overlap between these two unbiased approaches, predictions are more challenging for the low-abundant tissue types (Figure S5B). In summary, our untargeted ST data not only confirmed the effectiveness of our cross-species cell type mapping approach in predicting annotations and the overall robustness of our wheat root single-cell dataset but also provided high-confidence cell type/state-specific marker genes.

Our annotation approach, which links corresponding cell type clusters from different species and the validation of these cluster identities using ST, now also enables us to resolve inconsistencies in the annotations and strengthen predictions in the other datasets. For example, the *Tae*_2 cluster, validated to be the cortex cluster in our wheat soil-grown root dataset, has its best hits with *Osa*_cortex, *Zma*_Early Cortex II, and the sn*Tae*_Immature Pericycle Cells clusters. This would suggest that the annotation of sn*Tae*_Immature Pericycle Cells is questionable due to its conflict with all other lines of evidence. We thus re-annotate this cluster (Table S6; Figure S5A) to sn*Tae*_cortex for further analyses. Taken together, our cross-species cluster annotation approach not only enables comprehensive cell type annotation of our newly generated wheat atlas but also allows resolution of inconsistencies in publicly available datasets.[20]

Identification of robust evolutionarily conserved tissue-specific markers

We next aimed to define robust, tissue-specific markers that can be used for cluster annotation across different species by integrating the information in our wheat atlas and the public available scRNA-seq datasets in rice, maize, and *Arabidopsis*. This integration starts with the homogenization of input data through the reprocessing of these publicly available datasets with the same workflow used for our wheat scRNA-seq dataset (STAR Methods). The annotations from the original publications for these public datasets, resolved through previous analyses (Table S6; Figure S5A), were then transferred to the re-processed clusters using our statistical annotation transfer approach (Figures 3A–3D; Table S7). For each species in the analysis, all tissue-specific DEGs were determined and aggregated into experimentally validated major cell types, including root cap, epidermis, cortex, endodermis, pericycle, xylem and phloem, based on the cluster annotation. Tissue-specific DEGs

for all four species were then ranked on tissue specificity (q value). To aggregate the resulting ranks across species, we computed the average rank within each orthologous group, considering only the rank of the most tissue-specific (lowest q value) DEG in each species. First, markers are ranked based on the number of species in which they are conserved. Then, within each level of conservation, markers are further ranked on their average rank across species. For each of the orthologous groups, we report the final rank, which corresponds to the most tissue-specific DEG in each species. Specifically, for wheat DEGs that are triads (genes with one-to-one-to-one homeologs across the A, B, and D genomes), we report stability (there is no conflicting tissue specificity among expressed homeologs), specificity (the expressed homeologs are specific to only one tissue), as well as genome asymmetry (there is a bias in expression among the three homeologs), leveraging the homeolog expression pattern of allohexaploid wheat (STAR Methods). We found genome asymmetry within all clusters (Table S8; Figure S6; see STAR Methods for details). Among them, the root cap contains most triads with biased expression (5.4% of all triads in the wheat genome), while the cells in G2/M phase show the least (1.2%). Since cell type annotation is based on cluster DEGs, we extracted all 1,515 wheat triads (1:1:1 homeologs) that are cluster DEGs and found that 427 (28.2%) of them have at least two-thirds of their homeologs end up in different clusters, indicating homeolog expression divergence across cell types. These results (Table S9) not only summarize computationally inferred markers of four important plant species but also provide a convenient way for the plant single-cell community to identify potential robust orthologous markers in other, less studied organisms by focusing on orthologs that are conserved across different species or are monocot-specific. From this analysis, for example, we found 17 orthologous groups that are conserved across the four species in xylem (Figure 4A). One of these orthologous groups contains *XCP1*, which shows high xylem expression specificity across *Arabidopsis* (*At4g35350*), wheat (*TraesCS5A03G0388600/TraesCS5D03G0377000*), rice (*LOC_Os01g73980*), and maize (*Zm00001d035689*) scRNA-seq data (Figures 4B–4E). The wheat ortholog also showed xylem-specific expression in spatial data and is stable and balanced across three subgenomes (Figures 4F and 4G). Thus, we characterize *XCP1* as a robust, evolutionarily conserved marker for xylem tissues that is also stable and balanced among the homeologs in wheat. Although less specific in *Arabidopsis*, *DRN1* (*At2g45180*, *TraesCS1A03G0554700*, *LOC_Os04g46810*, *Zm00001d026163*) is an example of a conserved marker for cortex (Figure S7).

In addition to markers conserved across *Arabidopsis*, wheat, rice, and maize, we also focused on tissue-specific markers that are unique to the monocot clade with the rationale that, currently, orthologs to *Arabidopsis* markers are mostly used in monocots, while there might be more specific markers for the different root tissues in monocots (Table S9). For example, *TraesCS3A03G0974500/LOC_Os01g68589/Zm00001d012080* was found to be cortex-specific and is unique to monocot species, as the closest *Arabidopsis* homolog, *At5g61050*, showed no cortex specificity (Figures 4H–4L). Furthermore, the wheat ortholog also showed cortex-specific expression in our spatial data (Figures 4M and 4N). These results suggest that *TraesCS3A03G0974500/LOC_Os01g68589/Zm00001d012080* is a monocot-specific cortex marker. Another example of tissue-specific markers unique to the monocot clade is *TraesCS2D03G0321600/TraesCS2A03G0303700/TraesCS2B03G0423400*, *LOC_Os07g44550*, *Zm00001d006933* in the epidermis (Figure S8). In summary, our analyses provide 113 orthologous groups that are conserved across at least three species as a resource to the community in the form of predicted tissue-specific markers from which one can find those that are evolutionarily conserved across *Arabidopsis*, wheat, rice, and maize or, e.g., those unique to the monocot clade.

Cell type-specific GRN analysis retrieves both known and uncharacterized developmental regulators

Leveraging our annotated and validated wheat root atlas, we can now predict cell type-specific regulators and gene regulatory networks (GRNs). For this, we applied MINI-EX, which infers a set of cell type-specific GRNs using expression- and motif-based filtering and subsequently ranks transcription factor (TF) regulators on their predicted functional relevance.[53,54] GRNs derived solely from co-expression often yield numerous false positives, which can be mitigated by filtering putative target genes of a TF for those that contain the corresponding TF binding motif in their regulatory region. Given that motif mapping files for wheat are not available, we generated a *de novo* motif mapping file based on publicly available assay for transposase-accessible chromatin using sequencing (ATAC-seq) data in the wheat root.[55] This approach minimizes false positives by leveraging experimentally defined accessible chromatin regions, which are more likely to be bound by TFs and, thus, involved in transcriptional regulation (STAR Methods; Figure S9). Briefly, we processed the ATAC-seq data and identified peaks, which we considered regulatory regions for their nearest gene. We then mapped wheat TF motifs onto these regulatory regions, allowing us to associate the corresponding TFs and TF families to their potential target genes. With the ATAC-filtered motif mapping file, we employed MINI-EX and recovered cell type-specific regulons for which the TFs are orthologous to known regulators in *Arabidopsis*, including *VND1-4*[56] and *TMO5/T5L1*[57] for xylem, *DOF5.3*[58] for phloem, *RHL1*[59] for epidermis (root hair), and *MYB36*[60] for endodermis (Figure S10; Table S10). Given that our GRN analyses recover known regulators, it is likely that the unknown factors predicted in this analysis might also be tissue-specific regulators, making them interesting targets for further study in wheat. In order to provide similar GRN information for other key plant model species as a resource to the community, we ran MINI-EX on the reprocessed rice, maize, and *Arabidopsis* datasets (Figures S11–S13; Table S10) and performed a comparative analysis on the resulting GRNs. The TFs that were predicted to be the most functionally relevant for each of the major cell types were then compared between species using orthology. In order to both filter out spurious overlap of low-confidence regulators between species and to balance the number of regulators in the comparison, we only compared the top 50 most functionally relevant TFs using the MINI-EX ranking for each tissue and each species (Figure 5A; Table S11). This analysis allowed us to investigate the occurrence of a certain regulator in different cell types across four datasets and help reveal

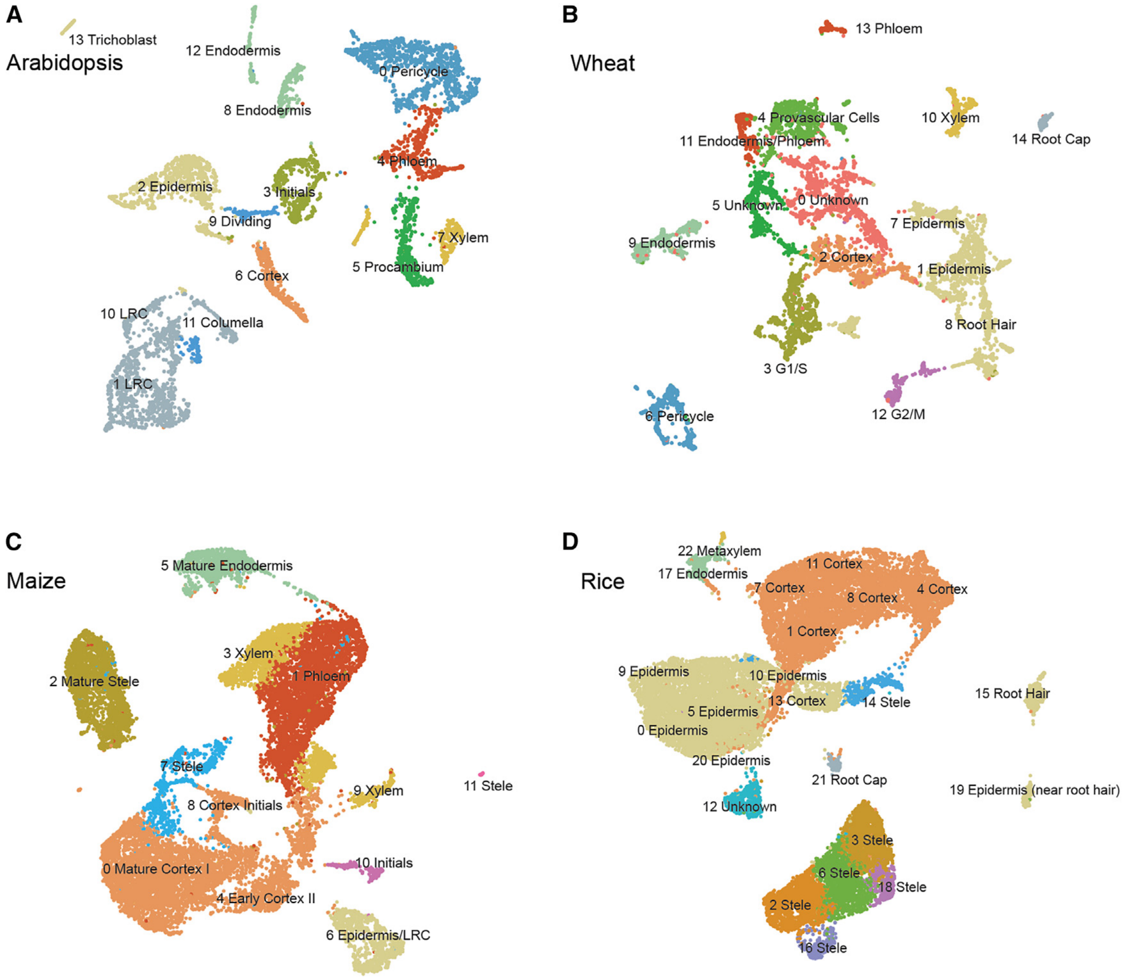

Figure 3. Reprocessed scRNA-seq datasets
(A) Reprocessed and transferred annotation of the *Arabidopsis* scRNA-seq dataset.[7]
(B) The wheat scRNA-seq dataset generated in this study.
(C) Reprocessed and transferred annotation of the maize scRNA-seq dataset.[24]
(D) Reprocessed and transferred annotation of the rice scRNA-seq dataset.[32]

both tissue-specific regulators conserved across all four species and regulators unique to the monocot clade (Table S11). For example, in xylem tissues, two regulators were found to be conserved across *Arabidopsis*, wheat, rice, and maize, including a known xylem developmental regulator, *EMB2749/NAC007/VND4* (*At1g12260*, *TraesCS7D03G0019800*, *LOC_Os04g45340*, *Zm00001d002828*) that causes ectopic deposition of secondary walls when overexpressed[61,62] (Figure 5B; Table S12), as well as an uncharacterized regulator, *ANAC002* (*At1g01720*, *TraesCS3A03G0950500*, *LOC_Os01g66120*, *Zm00001d038221*). Putative regulators unique to the monocot clade in xylem tissues include the uncharacterized NAC domain-containing protein 67-like (*TraesCS6B03G0176100*, *LOC_Os07g12340*, *Zm00001d019207*), NAC domain-containing protein 92-like (*TraesCS2D03G0214000*, *LOC_Os07g48550*, *Zm00001d022517*; *TraesCS4A03G0277300*, *LOC_Os03g21030*, *Zm00001d028995*), MYB4-like (*TraesCS2D03G0864800*, *LOC_Os04g43680*, *Zm00001d025864*), and ERF7-like (*TraesCS2D03G1207000*, *LOC_Os04g57340*, *Zm00001d001907*). Additionally, four root cap regulators—*ANAC081* (*At5g08790*), *WRKY17* (*At2g24570*), *WRKY26* (*At5g07100*), and *WRKY40* (*At1g80840*)—were found to be conserved across *Arabidopsis*, wheat, rice, and maize, where *ANAC081* (*At5g08790*) is a paralog of a known root cap-specific TF, *ANAC033/SOMBRERO* (*At1g79580*),[63] that plays a key role in root cap developmental programmed cell death (dPCD)-mediated physical defense mechanism limiting microbial invasion of the root.[64] Interestingly, WRKY proteins are well-known TFs that function in plant immunity to biotic stress, though they have not yet been studied at the cell type level. For example, *WRKY17* (*At2g24570*) has been shown to act as a negative regulator of basal resistance to the plant pathogen *Pseudomonas syringae*.[65] *WRKY40* (*At1g80840*) has been reported to interact with other WRKYs to function in plant responses to *P. syringae* and *Botrytis cinerea*.[66] Last, *WRKY26* (*At5g07100*) has been

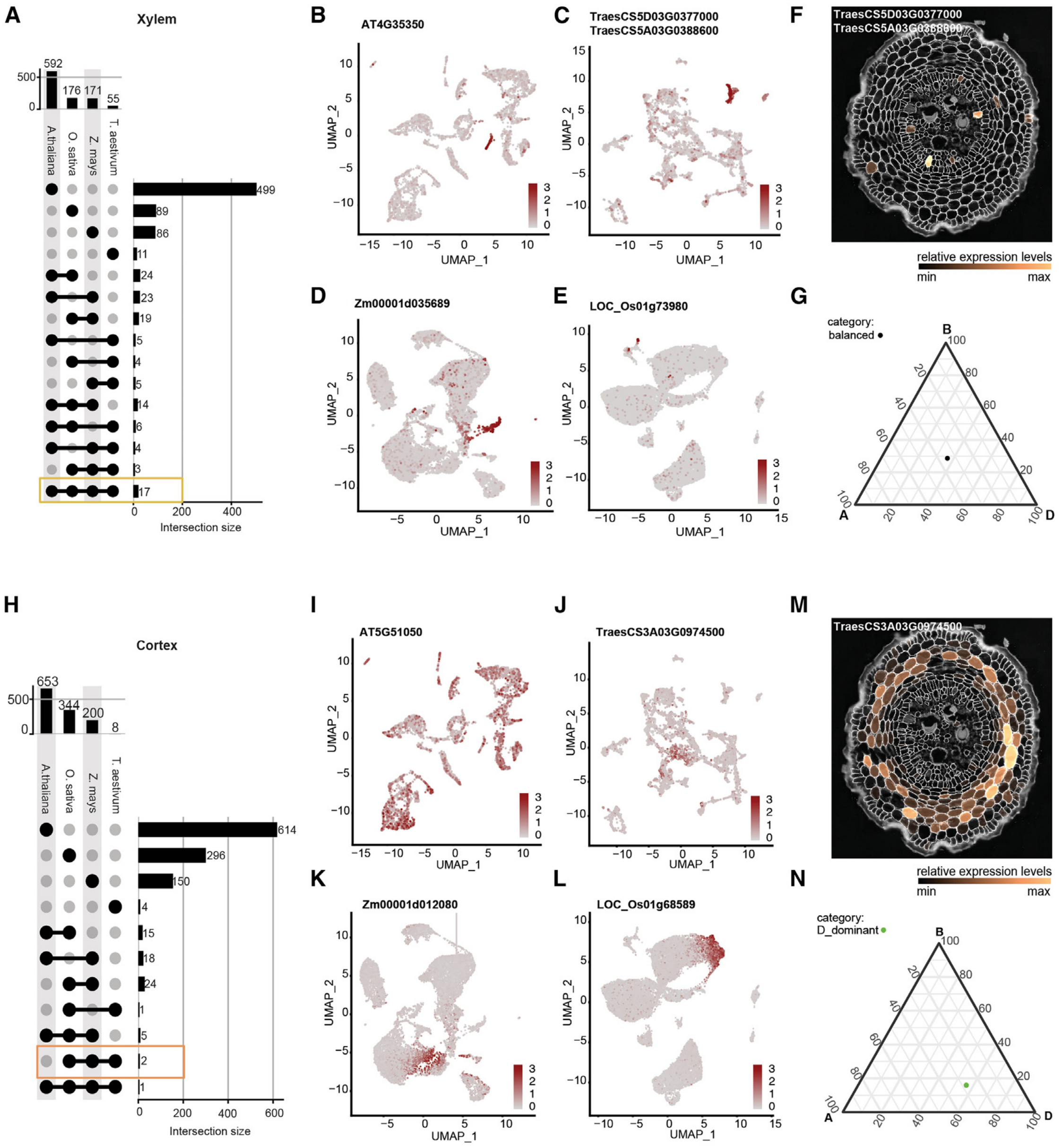

Figure 4. Tissue-specific markers conserved across *Arabidopsis*, wheat, rice, and maize or unique to the monocot clade

(A) UpSet plot showing the intersections of orthologous groups of xylem markers across *Arabidopsis*, wheat, rice, and maize.

(B–E) Feature plots of a xylem-specific marker across species.

(F and G) Spatial expression in STOmics Stereo-seq data (F) and ternary plot showing genome asymmetry information (G) of the same xylem-specific marker in the wheat root meristem.

(H) UpSet plot showing the intersections of orthologous groups of cortex markers across *Arabidopsis*, wheat, rice, and maize.

(I–L) Feature plots of a cortex-specific marker unique to monocots.

(M and N) Spatial expression in STOmics Stereo-seq data (M) and ternary plot showing genome asymmetry information (N) of the same cortex-specific marker in the wheat root meristem.

suggested to also play a role in basal immunity.[67] These three WRKY-type root cap regulators have been reported to play a role in plant microbial pathogen defense. This functional information not only supports the reliability and significance of the regulators identified in our analyses but also suggest that VND4-mediated secondary cell wall deposition in xylem and root cap dPCD-mediated defense mechanisms are cross-species conserved. In summary, these single-cell gene regulatory networks analyses predict numerous known and uncharacterized root developmental regulators, including those that are conserved across *Arabidopsis*, wheat, rice, and maize as well as those unique to the monocot clade. For the known and

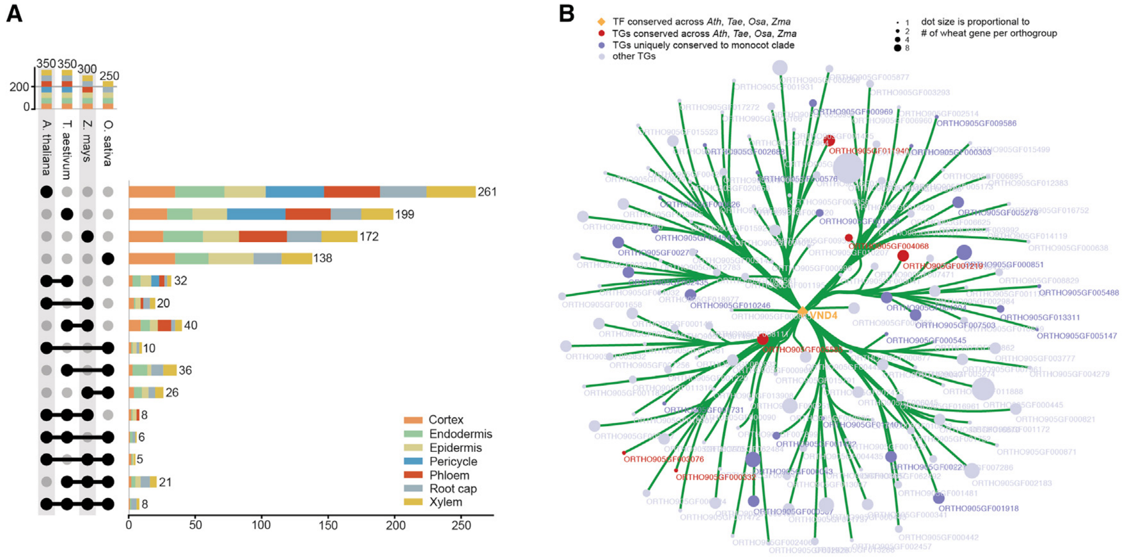

Figure 5. Cross-species overlap of predicted tissue-specific regulators

(A) UpSet plot showing the intersections of predicted regulators across *Arabidopsis*, wheat, rice, and maize.

(B) Cytoscape visualization of the GRN around wheat *VND4*. Dot size is proportional to the number of wheat genes in each orthologous group.

conserved regulators, the knowledge gained from *Arabidopsis* can now be transferred to less intensely studied crop species, while the uncharacterized regulators can be promising targets for future functional studies.

DISCUSSION

sc/snRNA-seq are powerful approaches for the unsupervised characterization of transcriptional variation in heterogeneous biological systems. However, separating heterogeneity in cell types or cell states from other sources of variation represents a challenge for both experimental design and computational analyses of scRNA-seq.[68] Moreover, given that clusters are, in essence, mathematical groupings, clusters only acquire biological relevance for further downstream analysis after undergoing detailed cell type annotation.[14,69] Although this has become a routine computational task for well-studied model systems like the root meristem of *Arabidopsis*, this is only possible by relying on decades of work identifying cell type and cell state marker genes.[39,40] In less studied organs and species, such elaborate resources are not readily available. ST promises to solve most of these issues in validating cell type annotations of non-model plant species in a high-throughput manner, as it avoids generating transgenic reporter lines.[17,18] Moreover, in contrast to animal cells, the cell wall of plant cells presents a unique advantage for defining cell boundaries during segmentation analysis. Despite these prospects, the real-life application of ST is still in its infancy in the plant field and by no means accessible to most research groups. Until this becomes a routine and affordable approach, cell type- and cell state-specific markers remain an important resource to the community for various molecular biology applications, including the interpretation of sc/snRNA-seq datasets. More specifically, the transferability between species of marker genes holds great importance when studying less-characterized plants and/or organs. By leveraging cross-species orthology, we were able to transfer cell type annotation from multiple available validated datasets to a newly generated dataset. Noteworthy is that the xylem cluster is the most identifiable and transcriptionally distinct cluster in all species used for this study. In comparison, the pericycle is one of the least clearly defined clusters in any of the datasets and is missing from our pipeline predictions. The existence of two separating phloem clusters (6 and 13) (Figure 1C), with only cluster 13 validated as phloem with spatial data, led us to speculate that cluster 6 could possibly be pericycle. This speculation was supported by two lines of evidence. First, by examining the top 25 DEGs of cluster 6, we noticed several genes (*TraesCS4B03G0262700*, *TraesCS6B03G0419800*, *TraesCS5B03G1121200*, *TraesCS4D03G0223700*, *TraesCS4A03G0535400*) with a clear expression in the pericycle layer in ST data, though not covering all individual pericycle cells and with some additional expression in phloem cells (Figure S4E). Further examination of these 5 genes showed that they are also low-ranking DEGs of cluster 13 and have enriched expression primarily in cluster 6 and somewhat in cluster 13 according to the UMAP visualization (Figure S4E). Second, two DEGs from cluster 6 (*TraesCS4D03G0748700* and *TraesCS4B03G0840800*) are orthologous to the *Arabidopsis* pericycle marker *At2g02130/PDF2.3*.[7] This suggests that cluster 6 is most likely the pericycle that shares transcriptomic features with phloem cells. We thus decided to manually annotate cluster 6 as the pericycle (Figure 1D).

Allohexaploid wheat presents a distinct challenge compared to diploid species such as *Arabidopsis* when it comes to defining cell type-specific markers due to the presence of three genomes. Several studies have looked into the expression of the gene homeologs at the tissue-specific level,[70] single-nucleus level,[20] and at single-cell level; e.g., in Chinese cabbage.[71] In our study, we took into account information of the subgenome

bias and the expression of homeologs when delineating the best possible conserved cell type-specific markers. We considered the best cell type markers to be expressed in the same cell types across all three genomes and assigned these as stable markers. This information contributes to the overall aim of highlighting the most specific cell type markers in our wheat root dataset but also defines the best possible conserved marker genes across monocot species in an unbiased manner considering the information currently available. In summary, the combination of a soil-grown wheat root transcriptome atlas, including experimental validation of the cluster identification on one hand with conserved marker identification and cell type-specific GRN on the other hand, provides an important resource for understanding root growth in monocot crops in general and wheat specifically.

Limitations of the study

Although our cross-species orthology-based annotation approach successfully annotated most cell clusters in a newly generated scRNA-seq dataset, the pipeline is only as good as the cell type annotations in the available datasets. For example, pericycle and phloem cluster annotations are missing from the rice dataset[32] either because those types of cells were not experimentally captured or because cluster identities were simply not resolved in the subsequent annotation and validation steps. Although ST approaches will solve most of the annotation challenges in years to come, current technologies have some clear limitations. For example, the untargeted ST approach we used has transcript diffusion as a major drawback. This can reduce resolution and compromise accuracy of the data, particularly in regions with small and heterogeneous cells, such as those found within the vascular tissues. Additionally, visualizing longitudinal sections to observe temporal expression levels comes with additional technical challenges for sectioning. However, the untargeted nature presents no restrictions on the number of genes analyzed compared to the targeted spatial approaches. The basic cell type annotation approach we present here can effectively resolve the majority of cell types in a newly generated single-cell atlas of a less-studied plant species and is therefore a useful addition to the toolbox of scientists venturing outside the well-studied *A. thaliana*.

RESOURCE AVAILABILITY

Lead contact

Requests for further information, resources, and reagents should be directed to and will be fulfilled by the lead contact, Bert De Rybel (bert.derybel@psb.vib-ugent.be).

Materials availability

This study did not generate new unique reagents.

Data and code availability

- Raw and processed data of the scRNA-seq and STOmics Stereo-Seq untargeted ST experiments have been deposited at NCBI and are publicly available as of the date of publication. Accession numbers are listed in the key resources table. The scRNA-seq data are accessible via our online browser tool (http://www.single-cell.be/plants).
- All code generated during this study is available at the GitHub repository and is publicly available as of the date of publication. Links to the code are listed in the key resources table.
- Any additional information required to reanalyze the data reported in this paper is available from the lead contact upon request.

ACKNOWLEDGMENTS

The authors would like to thank Li Pu, Jia Hui Khoo from BGI Research (Riga, Latvia) for help with the STOmics Stereo-seq experiments, and Sander Thierens for technical support for orthology inference. Part of this work was funded by a VLAIO grant from the Flemish Government (HBC.2019.2917 to Y.K., V.P., A.A., C.L.-M., Y.S., and B.D.R.) and by and the European Research Council (ERC StG TORPEDO; 714055 and ERC CoG PIPELINES, 101043257 to T.E. and B.D.R.). K.V. and B.D.R. received funding from EASI-Genomics TNA project PID7435. Y.S. and R.S. received funding from the Flemish Government under the “Onderzoeksprogramma Artificiële Intelligentie (AI) Vlaanderen” and from UGent project GOA.01G03524. J.S. was supported by BOF grant BOF24Y2019001901. L.P. is supported by FWO grant 01D16720. M.V.B. and K.V. are supported by FWO grant I000323N. We are thankful to the VIB Single Cell Core, VIB Flow Core Ghent, and VIB Nucleomics for support and access to the instrument park (vib.be/technologies). We also thank VIB Tech Watch for their support in setting up the cell enrichment and spatial transcriptomics technologies.

AUTHOR CONTRIBUTIONS

Y.K., V.P., and C.G. performed the experiments. Y.K., V.P., J.S., L.P., R.S., T.E., M.S.-S., and M.V.B. performed data analysis. Y.K. and M.V. performed the genome bias analysis. All authors discussed the results and contributed to the manuscript. C.L.-M., A.A., Y.S., and B.D.R. conceived and supervised the project. Y.K. and B.D.R. wrote the paper with input from all authors.

DECLARATION OF INTERESTS

The authors declare no competing interests.

STAR★METHODS

Detailed methods are provided in the online version of this paper and include the following:

- KEY RESOURCES TABLE
- EXPERIMENTAL MODEL AND STUDY PARTICIPANT DETAILS
  - Plant materials and growth conditions
- METHOD DETAILS
  - Preparation of root samples for scRNA-seq, library construction and sequencing
  - Pre-processing of raw scRNA-seq data
  - scRNA-seq quality controlled pipeline
  - Cross-species orthology-based cell type annotation pipeline
  - Preparation of root samples for STOmics stereo-seq
  - STOmics Stereo-seq data processing pipeline
  - Cell type/state-specific spatial expression pattern identification
  - Gene regulatory networks and wheat motif mapping
  - Marker stability
- QUANTIFICATION AND STATISTICAL ANALYSIS
  - Statistical model for computing genome asymmetry

SUPPLEMENTAL INFORMATION

Supplemental information can be found online at https://doi.org/10.1016/j.celrep.2025.115240.

Received: July 19, 2024
Revised: October 26, 2024
Accepted: January 7, 2025
Published: February 1, 2025

REFERENCES

1. Denyer, T., Ma, X., Klesen, S., Scacchi, E., Nieselt, K., and Timmermans, M.C.P. (2019). Spatiotemporal developmental trajectories in the Arabidopsis root revealed using high-throughput single-cell RNA sequencing. Dev. Cell *48*, 840–852.e5.
2. Jean-Baptiste, K., McFaline-Figueroa, J.L., Alexandre, C.M., Dorrity, M.W., Saunders, L., Bubb, K.L., Trapnell, C., Fields, S., Queitsch, C., and Cuperus, J.T. (2019). Dynamics of gene expression in single root cells of Arabidopsis thaliana. Plant Cell *31*, 993–1011.
3. Ryu, K.H., Huang, L., Kang, H.M., and Schiefelbein, J. (2019). Single-cell RNA sequencing resolves molecular relationships among individual plant cells. Plant Physiol. *179*, 1444–1456.
4. Shulse, C.N., Cole, B.J., Ciobanu, D., Lin, J., Yoshinaga, Y., Gouran, M., Turco, G.M., Zhu, Y., O'Malley, R.C., Brady, S.M., and Dickel, D.E. (2019). High-throughput single-cell transcriptome profiling of plant cell types. Cell Rep. *27*, 2241–2247.e4.
5. Turco, G.M., Rodriguez-Medina, J., Siebert, S., Han, D., Valderrama-Gómez, M.Á., Vahldick, H., Shulse, C.N., Cole, B.J., Juliano, C.E., Dickel, D.E., et al. (2019). Molecular mechanisms driving switch behavior in xylem cell differentiation. Cell Rep. *28*, 342–351.e4.
6. Zhang, T.-Q., Xu, Z.-G., Shang, G.-D., and Wang, J.-W. (2019). A single-cell RNA sequencing profiles the developmental landscape of Arabidopsis root. Mol. Plant *12*, 648–660.
7. Wendrich, J.R., Yang, B., Vandamme, N., Verstaen, K., Smet, W., Van de Velde, C., Minne, M., Wybouw, B., Mor, E., Arents, H.E., et al. (2020). Vascular transcription factors guide plant epidermal responses to limiting phosphate conditions. Science *370*, eaay4970.
8. Rich-Griffin, C., Stechemesser, A., Finch, J., Lucas, E., Ott, S., and Schäfer, P. (2020). Single-cell transcriptomics: a high-resolution avenue for plant functional genomics. Trends Plant Sci. *25*, 186–197.
9. Shaw, R., Tian, X., and Xu, J. (2021). Single-cell transcriptome analysis in plants: advances and challenges. Mol. Plant *14*, 115–126.
10. Cervantes-Pérez, S.A., Thibivillliers, S., Tennant, S., and Libault, M. (2022). Challenges and perspectives in applying single nuclei RNA-seq technology in plant biology. Plant Sci. *325*, 111486.
11. Denyer, T., and Timmermans, M.C.P. (2022). Crafting a blueprint for single-cell RNA sequencing. Trends Plant Sci. *27*, 92–103.
12. Minne, M., Ke, Y., Saura-Sanchez, M., and De Rybel, B. (2022). Advancing root developmental research through single-cell technologies. Curr. Opin. Plant Biol. *65*, 102113.
13. von der Mark, C., Minne, M., and De Rybel, B. (2024). Studying plant vascular development using single-cell approaches. Curr. Opin. Plant Biol. *78*, 102526.
14. Grones, C., Eekhout, T., Shi, D., Neumann, M., Berg, L.S., Ke, Y., Shahan, R., Cox, K.L., Gomez-Cano, F., Nelissen, H., et al. (2024). Best practices for the execution, analysis, and data storage of plant single-cell/nucleus transcriptomics. Plant Cell *36*, 812–828.
15. Xu, X., Crow, M., Rice, B.R., Li, F., Harris, B., Liu, L., Demesa-Arevalo, E., Lu, Z., Wang, L., Fox, N., et al. (2021). Single-cell RNA sequencing of developing maize ears facilitates functional analysis and trait candidate gene discovery. Dev. Cell *56*, 557–568.e6.
16. Li, C., Zhang, S., Yan, X., Cheng, P., and Yu, H. (2023). Single-nucleus sequencing deciphers developmental trajectories in rice pistils. Dev. Cell *58*, 694–708.e4.
17. Liu, Z., Kong, X., Long, Y., Liu, S., Zhang, H., Jia, J., Cui, W., Zhang, Z., Song, X., Qiu, L., et al. (2023). Integrated single-nucleus and spatial transcriptomics captures transitional states in soybean nodule maturation. Nat. Plants *9*, 515–524.
18. Li, X., Li, B., Gu, S., Pang, X., Mason, P., Yuan, J., Jia, J., Sun, J., Zhao, C., and Henry, R. (2024). Single-cell and spatial RNA sequencing reveal the spatiotemporal trajectories of fruit senescence. Nat. Commun. *15*, 3108.
19. Cole, B., Bergmann, D., Blaby-Haas, C.E., Blaby, I.K., Bouchard, K.E., Brady, S.M., Ciobanu, D., Coleman-Derr, D., Leiboff, S., Mortimer, J.C., et al. (2021). Plant single-cell solutions for energy and the environment. Commun. Biol. *4*, 962.
20. Zhang, L., He, C., Lai, Y., Wang, Y., Kang, L., Liu, A., Lan, C., Su, H., Gao, Y., Li, Z., et al. (2023). Asymmetric gene expression and cell-type-specific regulatory networks in the root of bread wheat revealed by single-cell multiomics analysis. Genome Biol. *24*, 65.
21. Wei, W.-Q., Li, S., Zhang, D., and Tang, W.-H. (2024). Single-Cell Transcriptomic Analysis Highlights Specific Cell Types of Wheat Manipulated by Fusarium graminearum Leading to Susceptibility. Preprint at bioRxiv. https://doi.org/10.1101/2024.06.08.598051.
22. Satterlee, J.W., Strable, J., and Scanlon, M.J. (2020). Plant stem-cell organization and differentiation at single-cell resolution. Proc. Natl. Acad. Sci. USA *117*, 33689–33699.
23. Marand, A.P., Chen, Z., Gallavotti, A., and Schmitz, R.J. (2021). A cis-regulatory atlas in maize at single-cell resolution. Cell *184*, 3041–3055.e21.
24. Ortiz-Ramírez, C., Guillotin, B., Xu, X., Rahni, R., Zhang, S., Yan, Z., Coqueiro Dias Araujo, P., Demesa-Arevalo, E., Lee, L., and Van Eck, J. (2021). Ground tissue circuitry regulates organ complexity in maize and Setaria. Science *374*, 1247–1252.
25. Li, X., Zhang, X., Gao, S., Cui, F., Chen, W., Fan, L., and Qi, Y. (2022). Single-cell RNA sequencing reveals the landscape of maize root tips and assists in identification of cell type-specific nitrate-response genes. Crops J. *10*, 1589–1600.
26. Tao, S., Liu, P., Shi, Y., Feng, Y., Gao, J., Chen, L., Zhang, A., Cheng, X., Wei, H., Zhang, T., and Zhang, W. (2022). Single-cell transcriptome and network analyses unveil key transcription factors regulating mesophyll cell development in maize. Genes *13*, 374.
27. Cao, Y., Ma, J., Han, S., Hou, M., Wei, X., Zhang, X., Zhang, Z.J., Sun, S., Ku, L., and Tang, J. (2023). Single-cell RNA sequencing profiles reveal cell type-specific transcriptional regulation networks conditioning fungal invasion in maize roots. Plant Biotechnol. J. *21*, 1839–1859.
28. Guillotin, B., Rahni, R., Passalacqua, M., Mohammed, M.A., Xu, X., Raju, S.K., Ramírez, C.O., Jackson, D., Groen, S.C., Gillis, J., and Birnbaum, K.D. (2023). A pan-grass transcriptome reveals patterns of cellular divergence in crops. Nature *617*, 785–791.
29. Sun, Y., Dong, L., Kang, L., Zhong, W., Jackson, D., and Yang, F. (2024). Progressive meristem and single-cell transcriptomes reveal the regulatory mechanisms underlying maize inflorescence development and sex differentiation. Mol. Plant *17*, 1019–1037.
30. Xu, X., Passalacqua, M., Rice, B., Demesa-Arevalo, E., Kojima, M., Takebayashi, Y., Harris, B., Sakakibara, H., Gallavotti, A., and Gillis, J. (2024). Large-scale single-cell profiling of stem cells uncovers redundant regulators of shoot development and yield trait variation. Preprint at bioRxiv. https://doi.org/10.1101/2024.03.04.583414.
31. Yuan, Y., Huo, Q., Zhang, Z., Wang, Q., Wang, J., Chang, S., Cai, P., Song, K.M., Galbraith, D.W., Zhang, W., et al. (2024). Decoding the gene regulatory network of endosperm differentiation in maize. Nat. Commun. *15*, 34.
32. Liu, Q., Liang, Z., Feng, D., Jiang, S., Wang, Y., Du, Z., Li, R., Hu, G., Zhang, P., Ma, Y., et al. (2021). Transcriptional landscape of rice roots at the single-cell resolution. Mol. Plant *14*, 384–394.
33. Wang, Y., Huan, Q., Li, K., and Qian, W. (2021). Single-cell transcriptome atlas of the leaf and root of rice seedlings. J. Genet. Genom. *48*, 881–898.
34. Wang, Z., Cheng, D., Fan, C., Zhang, C., Zhang, C., and Liu, Z. (2021). Cell type-specific differentiation between indica and japonica rice root tip responses to different environments based on single-cell RNA sequencing. Front. Genet. *12*, 659500.
35. Zhang, T.-Q., Chen, Y., Liu, Y., Lin, W.-H., and Wang, J.-W. (2021). Single-cell transcriptome atlas and chromatin accessibility landscape reveal differentiation trajectories in the rice root. Nat. Commun. *12*, 2053.
36. Zong, J., Wang, L., Zhu, L., Bian, L., Zhang, B., Chen, X., Huang, G., Zhang, X., Fan, J., Cao, L., et al. (2022). A rice single cell transcriptomic

CellPress
OPEN ACCESS

atlas defines the developmental trajectories of rice floret and inflorescence meristems. New Phytol. *234*, 494–512.

37. Zha, W., Li, C., Wu, Y., Chen, J., Li, S., Sun, M., Wu, B., Shi, S., Liu, K., Xu, H., et al. (2023). Single-Cell RNA sequencing of leaf sheath cells reveals the mechanism of rice resistance to brown planthopper (Nilaparvata lugens). Front. Plant Sci. *14*, 1200014.
38. Yao, J., Chu, Q., Guo, X., Shao, W., Shang, N., Luo, K., Li, X., Chen, H., Cheng, Q., and Mo, F. (2024). Spatiotemporal transcriptomic landscape of rice embryonic cells during seed germination. Dev. Cell *59*, 2320–2332.e5.
39. Birnbaum, K., Shasha, D.E., Wang, J.Y., Jung, J.W., Lambert, G.M., Galbraith, D.W., and Benfey, P.N. (2003). A gene expression map of the Arabidopsis root. Science *302*, 1956–1960.
40. Brady, S.M., Orlando, D.A., Lee, J.-Y., Wang, J.Y., Koch, J., Dinneny, J.R., Mace, D., Ohler, U., and Benfey, P.N. (2007). A high-resolution root spatiotemporal map reveals dominant expression patterns. Science *318*, 801–806.
41. Veselinyová, D., Mašlanková, J., Kalinová, K., Mičková, H., Mareková, M., and Rabajdová, M. (2021). Selected in situ hybridization methods: principles and application. Molecules *26*, 3874.
42. Nobori, T., Monell, A., Lee, T.A., Zhou, J., Nery, J., and Ecker, J.R. (2023). Time-resolved single-cell and spatial gene regulatory atlas of plants under pathogen attack. Preprint at bioRxiv. https://doi.org/10.1101/2023.04.10.536170.
43. Li, R., Wang, Z., Wang, J.-W., and Li, L. (2023). Combining single-cell RNA sequencing with spatial transcriptome analysis reveals dynamic molecular maps of cambium differentiation in the primary and secondary growth of trees. Plant Commun. *4*, 100665.
44. Xia, K., Sun, H.-X., Li, J., Li, J., Zhao, Y., Chen, L., Qin, C., Chen, R., Chen, Z., Liu, G., et al. (2022). The single-cell STOmics Stereo-seq reveals region-specific cell subtypes and transcriptome profiling in Arabidopsis leaves. Dev. Cell *57*, 1299–1310.e4.
45. Song, X., Guo, P., Xia, K., Wang, M., Liu, Y., Chen, L., Zhang, J., Xu, M., Liu, N., Yue, Z., et al. (2023). Spatial transcriptomics reveals light-induced chlorenchyma cells involved in promoting shoot regeneration in tomato callus. Proc. Natl. Acad. Sci. USA *120*, e2310163120.
46. Yang, N., Wang, Y., Luo, Y., Guo, X., Li, Y., Yan, J., Shao, W., Wei, W., Jackson, D., and Zhang, Z. (2023). A Dynamic transcriptome landscape of developing maize ear. The Plant Journal *116*, 1856–1870.
47. Lian, X., Zhong, L., Bai, Y., Guang, X., Tang, S., Guo, X., Wei, T., Yang, F., Zhang, Y., and Huang, G. (2024). Spatiotemporal transcriptomic atlas of rhizome formation in Oryza longistaminata. Plant Biotechnol. J. *22*, 1652–1668.
48. Tian, C., Du, Q., Xu, M., Du, F., and Jiao, Y. (2020). Single-nucleus RNA-seq resolves spatiotemporal developmental trajectories in the tomato shoot apex. Preprint at bioRxiv. https://doi.org/10.1101/2020.09.20.305029.
49. Chen, Y., Tong, S., Jiang, Y., Ai, F., Feng, Y., Zhang, J., Gong, J., Qin, J., Zhang, Y., Zhu, Y., et al. (2021). Transcriptional landscape of highly lignified poplar stems at single-cell resolution. Genome Biol. *22*, 1–22.
50. Cervantes-Pérez, S.A., Thibivilliers, S., Laffont, C., Farmer, A.D., Frugier, F., and Libault, M. (2022). Cell-specific pathways recruited for symbiotic nodulation in the Medicago truncatula legume. Mol. Plant *15*, 1868–1888.
51. Movahedi, S., Van Bel, M., Heyndrickx, K.S., and Vandepoele, K. (2012). Comparative co-expression analysis in plant biology. Plant Cell Environ. *35*, 1787–1798.
52. Chen, A., Liao, S., Cheng, M., Ma, K., Wu, L., Lai, Y., Qiu, X., Yang, J., Xu, J., Hao, S., et al. (2022). Spatiotemporal transcriptomic atlas of mouse organogenesis using DNA nanoball-patterned arrays. Cell *185*, 1777–1792.e21.
53. Ferrari, C., Manosalva Pérez, N., and Vandepoele, K. (2022). MINI-EX: integrative inference of single-cell gene regulatory networks in plants. Mol. Plant *15*, 1807–1824.
54. Staut, J., Manosalva Perez, N., Depuydt, T., Vandepoele, K., and Lukicheva, S. (2023). MINI-EX version 2: cell-type-specific gene regulatory network inference using an integrative single-cell transcriptomics approach. Preprint at bioRxiv. https://doi.org/10.1101/2023.12.24.573246.
55. Pei, H., Teng, W., Gao, L., Gao, H., Ren, X., Liu, Y., Jia, J., Tong, Y., Wang, Y., and Lu, Z. (2023). Low-affinity SPL binding sites contribute to subgenome expression divergence in allohexaploid wheat. Sci. China Life Sci. *66*, 819–834.
56. Kubo, M., Udagawa, M., Nishikubo, N., Horiguchi, G., Yamaguchi, M., Ito, J., Mimura, T., Fukuda, H., and Demura, T. (2005). Transcription switches for protoxylem and metaxylem vessel formation. Genes Dev. *19*, 1855–1860.
57. De Rybel, B., Adibi, M., Breda, A.S., Wendrich, J.R., Smit, M.E., Novák, O., Yamaguchi, N., Yoshida, S., Van Isterdael, G., and Palovaara, J. (2014). Integration of growth and patterning during vascular tissue formation in Arabidopsis. Science *345*, 1255215.
58. Miyashima, S., Roszak, P., Sevilem, I., Toyokura, K., Blob, B., Heo, J.O., Mellor, N., Help-Rinta-Rahko, H., Otero, S., Smet, W., et al. (2019). Mobile PEAR transcription factors integrate positional cues to prime cambial growth. Nature *565*, 490–494.
59. Karas, B., Amyot, L., Johansen, C., Sato, S., Tabata, S., Kawaguchi, M., and Szczyglowski, K. (2009). Conservation of lotus and Arabidopsis basic helix-loop-helix proteins reveals new players in root hair development. Plant Physiol. *151*, 1175–1185.
60. Kamiya, T., Borghi, M., Wang, P., Danku, J.M.C., Kalmbach, L., Hosmani, P.S., Naseer, S., Fujiwara, T., Geldner, N., and Salt, D.E. (2015). The MYB36 transcription factor orchestrates Casparian strip formation. Proc. Natl. Acad. Sci. USA *112*, 10533–10538.
61. Zhou, J., Zhong, R., and Ye, Z.-H. (2014). Arabidopsis NAC domain proteins, VND1 to VND5, are transcriptional regulators of secondary wall biosynthesis in vessels. PLoS One *9*, e105726.
62. Zhong, R., Lee, C., McCarthy, R.L., Reeves, C.K., Jones, E.G., and Ye, Z.-H. (2011). Transcriptional activation of secondary wall biosynthesis by rice and maize NAC and MYB transcription factors. Plant Cell Physiol. *52*, 1856–1871.
63. Fendrych, M., Van Hautegem, T., Van Durme, M., Olvera-Carrillo, Y., Huysmans, M., Karimi, M., Lippens, S., Guérin, C.J., Krebs, M., Schumacher, K., and Nowack, M.K. (2014). Programmed cell death controlled by ANAC033/SOMBRERO determines root cap organ size in Arabidopsis. Curr. Biol. *24*, 931–940.
64. Charura, N.M., Llamas, E., De Quattro, C., Vilchez, D., Nowack, M.K., and Zuccaro, A. (2023). Root cap cell corpse clearance limits microbial colonization in Arabidopsis thaliana. Preprint at bioRxiv. https://doi.org/10.7554/eLife.96266.2.
65. Journot-Catalino, N., Somssich, I.E., Roby, D., and Kroj, T. (2006). The transcription factors WRKY11 and WRKY17 act as negative regulators of basal resistance in Arabidopsis thaliana. Plant Cell *18*, 3289–3302.
66. Xu, X., Chen, C., Fan, B., and Chen, Z. (2006). Physical and functional interactions between pathogen-induced Arabidopsis WRKY18, WRKY40, and WRKY60 transcription factors. Plant Cell *18*, 1310–1326.
67. Kanofsky, K., Strauch, C.J., Sandmann, A., Möller, A., and Hehl, R. (2018). Transcription factors involved in basal immunity in mammals and plants interact with the same MAMP-responsive cis-sequence from Arabidopsis thaliana. Plant Mol. Biol. *98*, 565–578.
68. Stegle, O., Teichmann, S.A., and Marioni, J.C. (2015). Computational and analytical challenges in single-cell transcriptomics. Nat. Rev. Genet. *16*, 133–145.
69. Rusnak, B., Clark, F.K., Vadde, B.V.L., and Roeder, A.H.K. (2024). What Is a Plant Cell Type in the Age of Single-Cell Biology? It's Complicated. Annu. Rev. Cell Dev. Biol. *40*, 301–328.

70. Ramírez-González, R.H., Borrill, P., Lang, D., Harrington, S.A., Brinton, J., Venturini, L., Davey, M., Jacobs, J., Van Ex, F., and Pasha, A. (2018). The transcriptional landscape of polyploid wheat. Science *361*, eaar6089.
71. Sun, X., Feng, D., Liu, M., Qin, R., Li, Y., Lu, Y., Zhang, X., Wang, Y., Shen, S., Ma, W., and Zhao, J. (2022). Single-cell transcriptome reveals dominant subgenome expression and transcriptional response to heat stress in Chinese cabbage. Genome Biol. *23*, 262.
72. Zhu, T., Wang, L., Rimbert, H., Rodriguez, J.C., Deal, K.R., De Oliveira, R., Choulet, F., Keeble-Gagnère, G., Tibbits, J., Rogers, J., et al. (2021). Optical maps refine the bread wheat Triticum aestivum cv. Chinese Spring genome assembly. Plant J. *107*, 303–314.
73. Hao, Y., Hao, S., Andersen-Nissen, E., Mauck, W.M., Zheng, S., Butler, A., Lee, M.J., Wilk, A.J., Darby, C., Zager, M., et al. (2021). Integrated analysis of multimodal single-cell data. Cell *184*, 3573–3587.e29.
74. McGinnis, C.S., Murrow, L.M., and Gartner, Z.J. (2019). DoubletFinder: doublet detection in single-cell RNA sequencing data using artificial nearest neighbors. Cell Syst. *8*, 329–337.e4.
75. Korsunsky, I., Millard, N., Fan, J., Slowikowski, K., Zhang, F., Wei, K., Baglaenko, Y., Brenner, M., Loh, P.R., and Raychaudhuri, S. (2019). Fast, sensitive and accurate integration of single-cell data with Harmony. Nat. Methods *16*, 1289–1296.
76. Van Bel, M., Proost, S., Wischnitzki, E., Movahedi, S., Scheerlinck, C., Van de Peer, Y., and Vandepoele, K. (2012). Dissecting plant genomes with the PLAZA comparative genomics platform. Plant Physiol. *158*, 590–600.
77. Emms, D.M., and Kelly, S. (2019). OrthoFinder: phylogenetic orthology inference for comparative genomics. Genome Biol. *20*, 1–14.
78. Van der Walt, S., Schönberger, J.L., Nunez-Iglesias, J., Boulogne, F., Warner, J.D., Yager, N., Gouillart, E., and Yu, T.; scikit-image contributors (2014). scikit-image: image processing in Python. PeerJ *2*, e453.
79. Stringer, C., Wang, T., Michaelos, M., and Pachitariu, M. (2021). Cellpose: a generalist algorithm for cellular segmentation. Nat. Methods *18*, 100–106.
80. Palla, G., Spitzer, H., Klein, M., Fischer, D., Schaar, A.C., Kuemmerle, L.B., Rybakov, S., Ibarra, I.L., Holmberg, O., Virshup, I., et al. (2022). Squidpy: a scalable framework for spatial omics analysis. Nat. Methods *19*, 171–178.
81. Chiu, C.-L., and Clack, N. (2022). Napari: A Python multi-dimensional image viewer platform for the research community. Microsc. Microanal. *28*, 1576–1577.
82. Virshup, I., Rybakov, S., Theis, F.J., Angerer, P., and Wolf, F.A. (2021). anndata: Annotated data. Preprint at bioRxiv. https://doi.org/10.1101/2021.12.16.473007.
83. Evans, C.E.B., Arunkumar, R., and Borrill, P. (2022). Transcription factor retention through multiple polyploidization steps in wheat. G3 *12*, jkac147.
84. Hitz, B.C., Lee, J.-W., Jolanki, O., Kagda, M.S., Graham, K., Sud, P., Gabdank, I., Strattan, J.S., Sloan, C.A., and Dreszer, T. (2023). The ENCODE uniform analysis pipelines. Preprint at bioRxiv. https://doi.org/10.1101/2023.04.04.535623.
85. Yu, G., Wang, L.-G., and He, Q.-Y. (2015). ChIPseeker: an R/Bioconductor package for ChIP peak annotation, comparison and visualization. Bioinformatics *31*, 2382–2383.
86. Wang, Q., Li, M., Wu, T., Zhan, L., Li, L., Chen, M., Xie, W., Xie, Z., Hu, E., Xu, S., and Yu, G. (2022). Exploring epigenomic datasets by ChIPseeker. Curr. Protoc. *2*, e585.
87. Grant, C.E., Bailey, T.L., and Noble, W.S. (2011). FIMO: scanning for occurrences of a given motif. Bioinformatics *27*, 1017–1018.
88. Weirauch, M.T., Yang, A., Albu, M., Cote, A.G., Montenegro-Montero, A., Drewe, P., Najafabadi, H.S., Lambert, S.A., Mann, I., Cook, K., et al. (2014). Determination and inference of eukaryotic transcription factor sequence specificity. Cell *158*, 1431–1443.
89. Medina-Rivera, A., Defrance, M., Sand, O., Herrmann, C., Castro-Mondragon, J.A., Delerce, J., Jaeger, S., Blanchet, C., Vincens, P., Caron, C., et al. (2015). RSAT 2015: regulatory sequence analysis tools. Nucleic Acids Res. *43*, W50–W56.
90. Camacho, C., Coulouris, G., Avagyan, V., Ma, N., Papadopoulos, J., Bealer, K., and Madden, T.L. (2009). BLAST+: architecture and applications. BMC Bioinf. *10*, 1–9.
91. Van Bel, M., Silvestri, F., Weitz, E.M., Kreft, L., Botzki, A., Coppens, F., and Vandepoele, K. (2022). PLAZA 5.0: extending the scope and power of comparative and functional genomics in plants. Nucleic Acids Res. *50*, D1468–D1474.
92. Tian, F., Yang, D.-C., Meng, Y.-Q., Jin, J., and Gao, G. (2020). PlantRegMap: charting functional regulatory maps in plants. Nucleic Acids Res. *48*, D1104–D1113.
93. Allison, D.B., Gadbury, G.L., Heo, M., Fernández, J.R., Lee, C.-K., Prolla, T.A., and Weindruch, R. (2002). A mixture model approach for the analysis of microarray gene expression data. Comput. Stat. Data Anal. *39*, 1–20.

CellPress
OPEN ACCESS

STAR★METHODS

KEY RESOURCES TABLE

REAGENT or RESOURCE	SOURCE	IDENTIFIER
**Biological samples**		
Triticum aestivum cultivar Chinese Spring	BASF Innovation Center Ghent	N/A
**Chemicals, peptides, and recombinant proteins**		
CELLULASE ONOZUKA^{TM}RS	Yakult	Cat# L0011
CELLULASE ONOZUKA^{TM}R-10	Yakult	Cat# L0012
MACEROZYME R-10	Duchefa	Cat# M8002
Pectolyase Y23 G	Kyowa	Cat# L001794
$CaCl_2$	Sigma	Cat# C5670
KCl	Sigma	Cat# 7447-40-7
MES Monohydrate	Duchefa	Cat# M1503
BSA	Sigma-Aldrich	Cat# A7906
beta-mercaptoethanol	Sigma	Cat# 63689
D-Mannitol	Sigma	Cat# M1902
Propidium iodide	sigma-aldrich	Cat# P4170
Calcein, AM	Invitrogen	Cat# C1430
Tissue-Tek OCT	Sakura	Cat# 4583
FluorescentBrightener 28	Sigma	Cat# F3543-5G
SSC buffer	Thermo	Cat# AM9770
RNase Inhibitor	NEB	Cat# M0314L
**Critical commercial assays**		
70 μm Cell Strainer	Falcon	Cat# 352340
Chromium Single Cell 3′ GEM, Library & Gel Bead Kit	10× Genomics	Cat# 1000268
**Deposited data**		
Single-cell datasets generated in this study.	NCBI's Gene Expression Omnibus	GEO id: GSE270342
Spatial transcriptomics datasets generated in this study.	NCBI's Gene Expression Omnibus	GEO id: GSE271725
GitHub workbook general manuscript codes	GitHub	https://github.com/VIB-PSB/wheat_root_atlas; https://doi.org/10.5281/zenodo.14524519
GitHub workbook annotation pipeline	GitHub	https://github.com/VIB-PSB/wheat_root_atlas; https://doi.org/10.5281/zenodo.14524587
Arabidopsis data	Wendrich et al.[7]	GEO id: GSE141730
Rice data	Liu et al.[32]	GEO id: GSE146035
Maize data	Ortiz-Ramírez et al.[24]	GEO id: GSE172302
**Software and algorithms**		
Cellranger	10x Genomics	Version 6.0.0
Seurat	Hao et al.[73]	Version 4.2.0
DoubletFinder	McGinnis et al.[74]	Version 2.0.3
Harmony	Korsunsky et al.[75]	Version 0.0.1
SAW	BGI Research	Version 1.0
Genstat	VSN International	Version 23
MINI-EX	Ferrari et al.[53]	Version 2.2

(*Continued on next page*)

Continued

REAGENT or RESOURCE	SOURCE	IDENTIFIER
ENCODE ATAC-seq pipeline	Hitz et al.[84]	Version 2.2.2
ChIPseeker	Yu et al.[85]	N/A
FIMO	Grant et al.[87]	Version 5.5.5
RSAT	Medina-Rivera et al.[89]	N/A
BLAST+	Camacho et al.[90]	Version 2.6.0
OrthoFinder	Emms et al.[77]	Version 2.5.3
napari	Chiu and Clack[81]	Version 0.4.16
anndata	Virshup et al.[82]	Version 0.8.0
R	R Core Team, 2023	Version 4.3.1
Python (STOmics Stereo-seq data processing)	python.org	Version 3.10.10
Python (annotation transfer, conserved marker analysis & GRN regulon analysis)	python.org	Version 3.8.0
Other		
STOmics Stereo-seq capture chips	BGI	N/A
DNBSEQ-Tx sequencer	BGI	N/A
CIS-BP	Weirauch et al.[88]	Build 2.00
Plaza Monocots	Van Bel et al.[91]	Version 5.0
PlantTFDB	Tian et al.[92]	N/A

EXPERIMENTAL MODEL AND STUDY PARTICIPANT DETAILS

Plant materials and growth conditions

The Chinese Spring Wheat roots used for both scRNA-seq and STOmics Stereo-seq spatial transcriptomics were cut from 15-day-old seedlings. Seed stock was obtained from BASF Innovation Center Ghent. Seeds were pre-soaked in Milli-Q water for 4 days in the dark at 4°C, followed by 10-min treatment at 50°C in a water bath. Seeds were then transferred onto an MS (Murashige and Skoog) medium plate, grown in a tissue culture room (24°C, 06.00–22.00 light). After germination, seeds were transferred to soil (Jiffy) in a growth room (20°C, 06.00–22.00 light).

METHOD DETAILS

Preparation of root samples for scRNA-seq, library construction and sequencing

A total of 100 root tips were harvested per sample (~0.5 cm from root tip) from all available sources (primary, lateral root) and digested for 1.5 h in enzyme buffer (0.4 M mannitol, 20 mM MES, 20 mM KCl, 1.25% Cellulase RS (Yakult), 1.25% Cellulase R10 (Yakult), 0.3% Macerozyme R10 (Yakult), 0.12% Pectolyase Y-23 (Yakult), 10 mM $CaCl_2$, 0.1% BSA, 0.018% beta-mercaptoethanol, pH = 5.7, the enzyme buffer was pre-heated at 60°C for 10 min, cool down to room temperature before use) with gentle shaking at room temperature. The protoplasts were filtered with a cell strainer (70 μm diameter, Falcon REF #352340), collected by centrifugation (200 g, 6 min), washed with 8% mannitol, filtered again with a cell strainer (70 μm diameter, Falcon REF #352340) and collected by centrifugation. The pellets were resuspended in 255 μl 8% mannitol and 45 μl levitation buffer (Levitas Bio). PI (Propidium iodide) and Calcein were added to the suspension. Cells were sorted using the LeviCell magnetic sorter (Levitas Bio). The purified cell suspension was then loaded onto a Chromium Single Cell 3′ GEM, Library & Gel Bead Kit (V3.1 chemistry, 10X Genomics) according to the manufacturer's instructions. Libraries were sequenced on an Illumina NovaSeq 6000 instrument following recommendations of 10X Genomics at the VIB Nucleomics Core (VIB, Leuven).

Pre-processing of raw scRNA-seq data

The raw scRNA-seq dataset was initially processed using Cell Ranger 6.0.0 (10X Genomics). These pipelines include demultiplexing raw scRNA-seq data, aligning reads to the genome and generating gene-cell matrices. The genome and GTF files of *Triticum aestivum* were downloaded from IWGSC v2.1,[72] with the chloroplast and mitochondrial genomes appended (>MH051715.1 *Triticum aestivum* cultivar Chinese Spring chloroplast, complete genome; >MH051716.1 *Triticum aestivum* cultivar Chinese Spring mitochondrion, complete genome) (Run 'cellranger mkref' with "-genome, -fasta and -genes" to build the genome reference. Run 'cellranger count' with "-id, transcriptome, -fastqs, -sample" to generate single-cell gene counts). The resulting filtered_gene_bc_matrices were used as the input for downstream analyses. For Arabidopsis,[7] maize,[24] and rice,[32] the filtered_gene_bc_matrices were downloaded according to the original literature.

scRNA-seq quality controlled pipeline

For wheat, Arabidopsis, maize, and rice, the gene-cell matrices (filtered_gene_bc_matrices) were loaded into the Seurat package (v 4.2.0)[73] and were individually processed. Quality control pipelines include keeping genes that are present in at least 3 cells and cells containing at least 500 genes and 800 UMIs; for maize, cells containing at least 1,200 genes and 3,500 UMIs are kept. Cells with more than 10% of mitochondrial reads and 5% of chloroplast reads were removed. Each single-cell dataset was then normalized using SCTransform from the Seurat package. 3000 variable features were returned for principal component analysis (PCA) and 30 PCs were used for clustering using FindNeighbors and FindClusters function with resolution = 0.3. Each pre-processed Seurat object was then used as input for doublet removal using DoubletFinder.[74] After filtering, three datasets were merged in wheat, SCTransformed and integrated using Harmony.[75] The same procedure was followed for the three replicates of Arabidopsis, nine replicates for maize, and two replicates for rice, respectively. The resultant integrated datasets were clustered using harmony dimensionality reductions. Clusters were identified using FindNeighbors and FindClusters with resolution = 0.24 for wheat, Arabidopsis, and maize, while fine-tuned for rice using the resolution = 1, and visualized using UMAP. Marker genes were identified using "FindAllMarkers" with logfc.threshold = 0.25, min.pct = 0.1.

Cross-species orthology-based cell type annotation pipeline

The cell type annotation source was based on DEG-annotation provided from the original paper,[7,20,24,32] the cluster annotation to be transferred includes the newly generated wheat atlas as well as reprocessed Arabidopsis, maize, and rice. Firstly, for the newly generated wheat atlas and re-processed datasets, all cluster marker genes were generated and filtered by the threshold avglog2FC > 0.5 and FDR <0.05; while for the original datasets, cluster marker genes and corresponding annotations were kept the way the original literatures defined/described. For each cross-species comparison, cluster-specific DEG were first collapsed into their orthologous groups (i.e., differentially expressed groups). These orthologous groups were constructed as part of a custom PLAZA build[76] using OrthoFinder[77] with the species *Amborella trichopoda*, *Arabidopsis thaliana*, *Medicago truncatula*, *Oryza sativa*, *Setaria italica*, *Solanum lycopersicum*, *Triticum aestivum*, *Vitis vinifera*, and *Zea mays*. In contrast, for intra-species comparisons, the overrepresentation analysis detailed below was done using gene IDs instead of orthologous groups (below, DEG refers to both differentially expressed genes and groups). A background distribution of DEG sets, drawn from all DEG that are observed as a marker for any cluster, is constructed for each set of cluster-specific DEG. All clusters of the reference dataset are then compared to all clusters of the query dataset. For each comparison, a fold enrichment value is calculated as the number of DEG that overlap between the real reference and query cluster, divided by the median DEG overlap between the query cluster and the reference background DEG sets. A *p* value is calculated as the number of times the overlap with the background sets is larger than or equal to the real overlap, divided by the number of background sets. After comparing all query and reference clusters, the Benjamini-Hochberg procedure was applied to adjust *p* values for multiple testing (q values). The annotation of the best (lowest q value) corresponding reference cluster is then transferred, given that the overlap is significant (q value >0.05). The code used for this annotation transfer approach is available through GitHub (https://github.com/VIB-PSB/cross_species_annotation_transfer).

Preparation of root samples for STOmics stereo-seq

Roots were harvested fresh and embedded in pre-cooled OCT (Sakura) and stored at −80°C until processed. All following steps were performed according to the protocols from[44,52] with small modifications. In brief, the pre-frozen root tissues in OCT were cross-sectioned at 10 μm thickness using a Leica CM1950 cryostat. STOmics Stereo-seq chip was pretreated with 0.01% poly-lysine for 10 min at room temperature before use. Tissue sections were adhered to the STOmics Stereo-seq chip surface and incubated on a slide warmer at 37°C for 8 min. Then, tissues were fixed in methanol and incubated at −20°C for 30 min and afterward stained with Fluorescent Brightener 28 (FB) and Qubit ssDNA. Imaging was performed with a Motic fluorescence microscope. Then the tissue sections were permeabilized at 37°C for 12 min followed by a washing step with 0.1x SSC buffer (Thermo, AM9770) containing 0.05 U/μL RNase inhibitor (NEB, M0314L). RNA was reverse transcribed for 3 h at 42°C, tissue sections were washed twice with 0.1x SSC buffer and digested with Tissue Removal buffer at 55°C for 10 min cDNA release mix was added and the cDNA was released during an overnight treatment at 55°C cDNA was purified using DNA Cleanup Beads AMPure(R) XP (Agencourt), and amplified using PCR. A total of 30 ng of DNA were used for fragmentation, performed at 55°C for 10 min. Fragmentation products were amplified using PCR, purified using the DNA Cleanup Beads AMPure(R) XP, and sequenced (paired-end 100 bp) on an MGI-DNBSEQ-Tx sequencer.

STOmics Stereo-seq data processing pipeline

Quality control, genome alignment and quantification of gene expression of the sequencing data were done using STOmics Analysis Workflow (SAW) (https://github.com/BGIResearch/SAW). Downstream analyses were done in R (4.3.1) and Python (3.10.10). For each root section, the corresponding cell-wall image was manually cropped around it (2000 × 2000 pixels) and the corresponding GEM was filtered accordingly. This ensured optimal alignment and faster processing. Cell-wall images were preprocessed using scikit-image[78] to remove background debris and enhance quality. Cells were automatically defined using Squidpy with the cellpose method,[79,80] as well as annotated using napari (0.4.16).[81] Protein-coding transcripts from the GEM files were assigned to their corresponding cell, and were summed per gene, resulting in a cell-by-gene count matrix. Data was stored in an AnnData object (0.8.0)[82] and gene expression within each cell was visualized using napari.

Cell type/state-specific spatial expression pattern identification

The spatial expression patterns of major cell types (epidermis, cortex, endodermis, pericycle, xylem, phloem) were assessed visually based on the anatomical features of the concentric cell layers of the root (Figure 2A). For example, enriched expression at the first layer (from outer to inner) of the section is considered as the spatial expression pattern of epidermis. Noteworthy, for provascular cells, enriched expression at the center of the section is expected as the dead hollow metaxylem attracts diffused signals from the provascular region (Figure 2A). For the spatial expression pattern of G1/S and G2/M phase, a patchy expression in most cell types across a section is expected as not all cells are undergoing cell division.

Gene regulatory networks and wheat motif mapping

GRNs were inferred using MINI-EX (v2.2),[53,54] using the gene-to-cell count matrix extracted from the final Seurat object, along with the corresponding clusters and cell type annotations. While MINI-EX natively incorporates databases with TF information and pre-computed motif mapping results for Arabidopsis, rice, and maize, a dedicated database was established for wheat, as described below. TFs and their families were obtained from[83] with IDs converted from IWGSC v1.1 to v2.1 using the IWGSC conversion list. Unlike motif mapping results in the native databases, which rely on predefined rules for the identification of regulatory regions, our approach in wheat utilizes accessible chromatin regions obtained from an ATAC-seq study in wheat roots[55] to minimize false positives. ATAC-seq data were processed using the ENCODE ATAC-seq pipeline v2.2.2,[84] and peaks (‘overlap.optimal_peak.narrowPeak‘) were considered as regulatory regions for their nearest gene using ChIPseeker.[85,86] A few genes with regulatory regions over 10 kb were excluded. TF motifs were mapped onto regulatory regions using FIMO[87] from MEME v5.5.5 (motifs were converted to MEME format using ‘matrix2meme′ with a background calculated on all regulatory regions using ‘fasta-get-markov‘). Wheat motifs were obtained from CIS-BP build 2.00,[88] with redundant motifs removed using ‘compare-matrices‘ from RSAT[89] (normalize correlation $\geq$1). Corresponding TFs with IWGSC v2.1 IDs were retrieved using ‘blastp‘ (BLAST+ 2.6.0)[90] between the full sequences of CIS-BP TFs and IWGSC peptides, selecting the best hit for each TF based on sequential criteria (lowest ‘evalue $\leq$ 1e-50′, highest ‘bitscore‘, highest ‘pident $\geq$90′, same chromosome in IDs, random). Wheat gene ontology for Biological Process (BP) was downloaded from Plaza Monocots 5.0.[91] Furthermore, we extended the native TF information for rice and maize by adding orthologs of known TFs in wheat, rice, maize and Arabidopsis. Known TFs were obtained from PlantTFDB[92] and orthologous TFs were added based on the PLAZA Integrative Orthology ensemble methodology (requiring at least two evidence types), using the same custom PLAZA build described in the orthology-based annotation Methods section. For all species, MINI-EX was then run using default parameters, without providing GO terms of interest. Only in wheat, the “topMarkers” parameter is increased from 700 to 2100 to account for wheat’s increased ploidy.

Marker stability

A total of 18,364 High-confidence homeolog triads for 55,092 genes were extracted from existing data,[70] among which 15,874 of them have detectable expression (UMI >0) in our scRNA-seq data. A triad is considered a stable marker if its expressed homeologs are DEG of the same tissue types and is considered a specific marker if its expressed homeologs are DEG of only one same tissue type.

QUANTIFICATION AND STATISTICAL ANALYSIS

Statistical model for computing genome asymmetry

A log-linear regression model of the form $y = \mu + \text{replicate} + \text{cluster*subgenome} + \varepsilon$, with a log link function, as implemented in Genstat (version 23, VSN International) was fitted to the average UMI counts. The dispersion parameter for the variance of the response was estimated from the residual mean square of the fitted model. Likelihood tests were used to assess the significance of the cluster. subgenome interaction term, by dropping this term from the full model. T-statistics were used to assess the significance of subgenome effects (on the logit transformed scale) by pairwise comparisons to a particular subgenome set as reference level. For example, we define A-dominant or A-suppressed as A-homeologs that are significantly higher (difference >0, FDR <0.05) or lower (difference <0, FDR <0.05) expressed than B-homeologs and D-homeologs. The false discovery rates (FDRs) were estimated by modeling the p values as a 2-component mixture of Uniform and Beta densities,[93] as implemented in GenStat v23; default parameter settings were used to estimate p0, the proportion of features that are truly null.

Cell Reports, Volume 44

Supplemental information

A single-cell and spatial wheat root atlas

with cross-species annotations delineates

conserved tissue-specific marker genes and regulators

Yuji Ke, Vincent Pujol, Jasper Staut, Lotte Pollaris, Ruth Seurinck, Thomas Eekhout, Carolin Grones, Maite Saura-Sanchez, Michiel Van Bel, Marnik Vuylsteke, Andrea Ariani, Christophe Liseron-Monfils, Klaas Vandepoele, Yvan Saeys, and Bert De Rybel

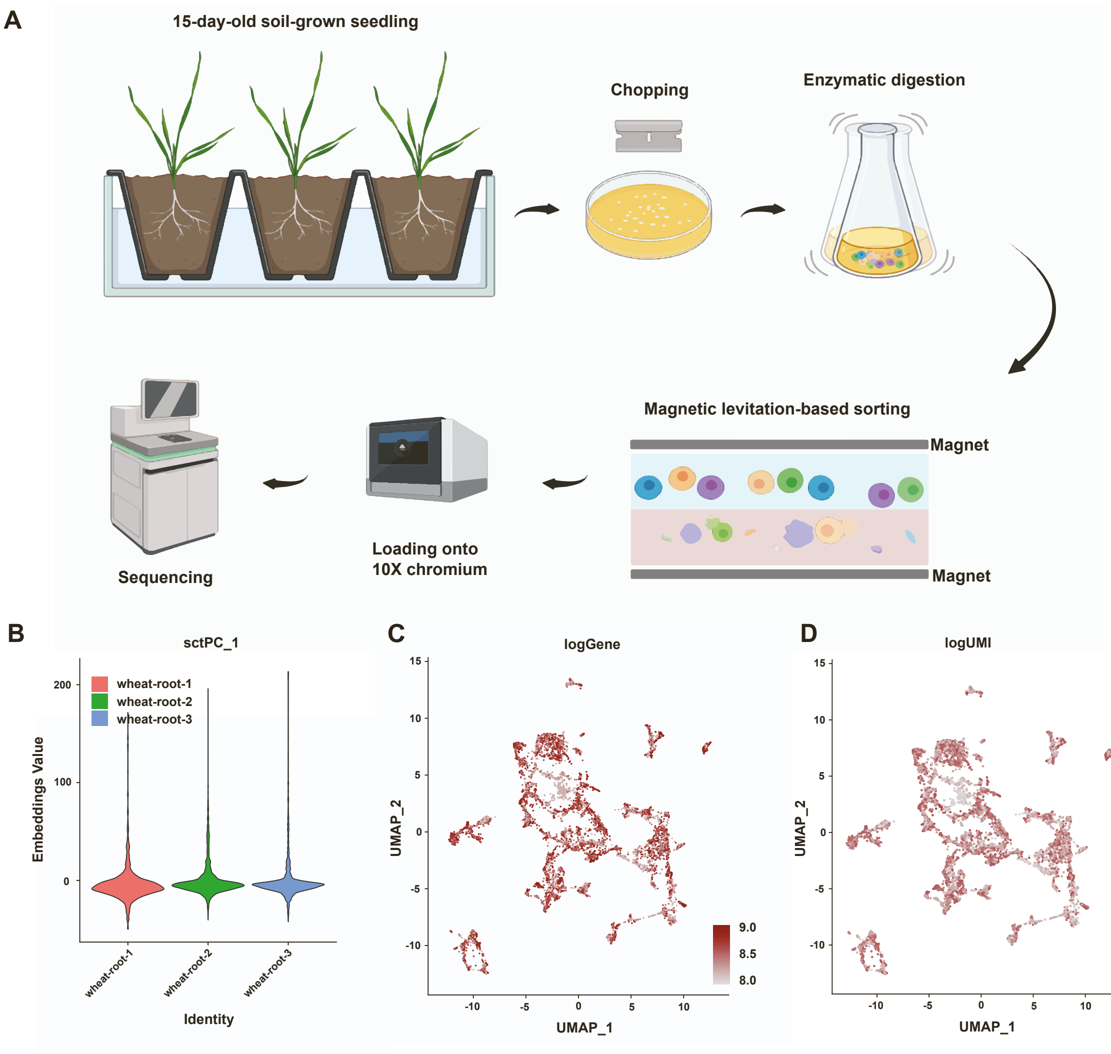

Figure S1. Experimental workflow and quality control of the wheat root apical meristem single-cell RNA-seq. (A) Overview of wheat scRNA-seq experimental workflow. Protoplasts were isolated from 5-mm root tips of 15-day soil-grown Chinese Spring wheat, cleaned using a magnetic sorter, loaded onto the 10x Genomics platform followed by high-throughput sequencing. (B) Violin plot showing the distribution of gene expression values for a specific principal component (sctPC_1) across three replicates. (C) Integrated UMAP showing Gene content distribution across cells. (D) Integrated UMAP showing UMI content distribution across cells.

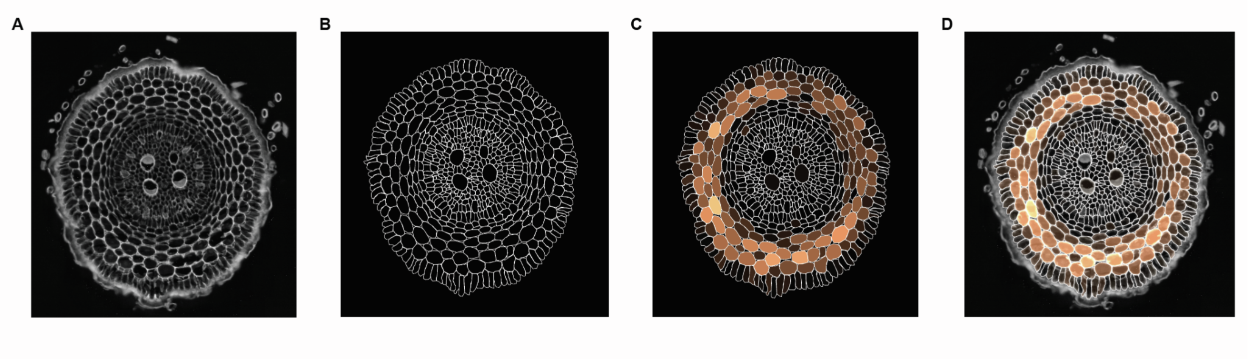

Figure S2. Stereo-Seq image processing. (A) Raw image of a root cross section stained with FB. (B) Manual segmentation of the root cross section. (C) Expression pattern of a cortex marker on segmented cells. (D) Overlay of expression pattern of a cortex marker, segmented cells, and raw image of the root section.

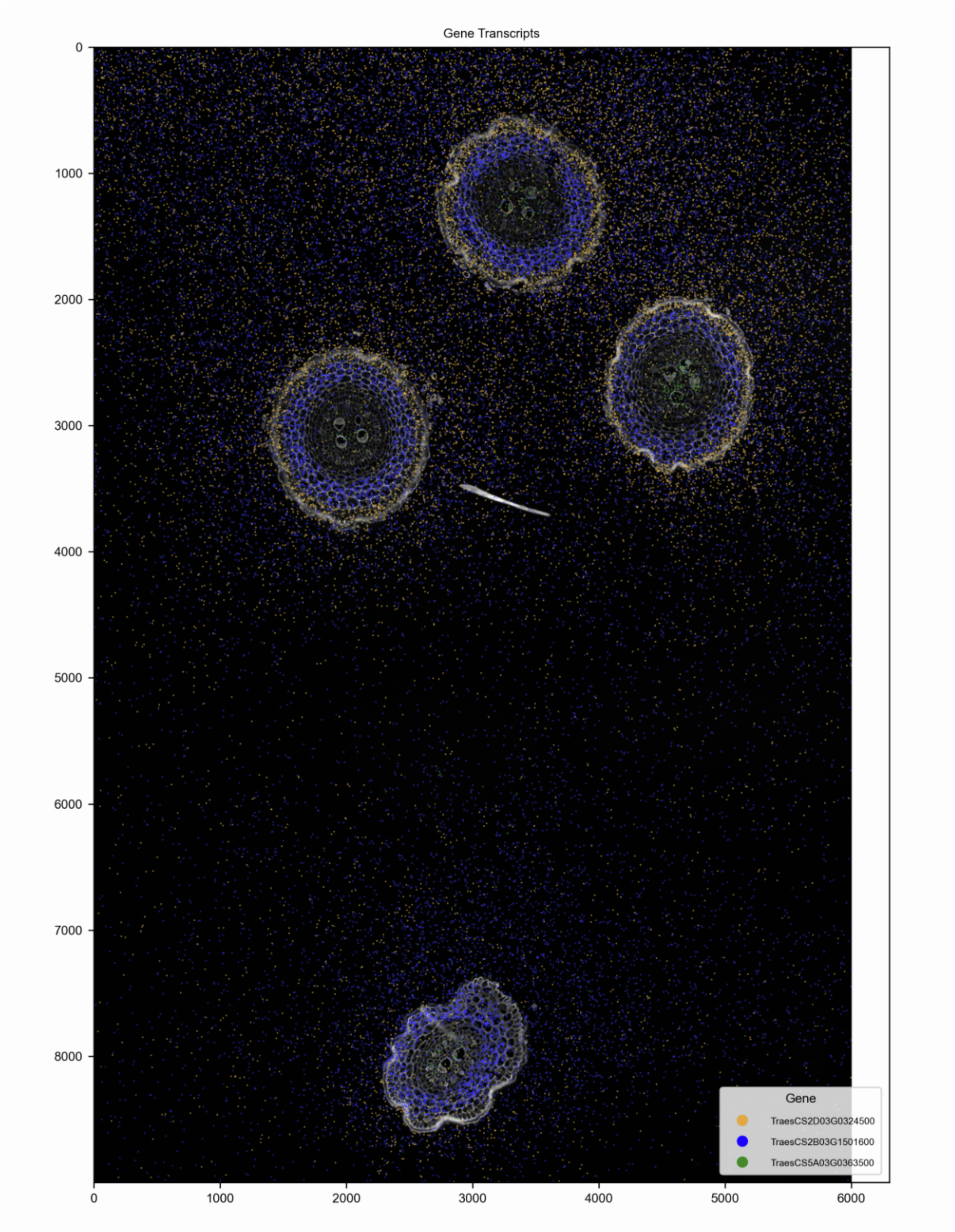

Figure S3. Expression pattern of an epidermis, cortex, and provascular cells marker gene on unsegmented Stereo-seq section image. Dots outside the tissue section indicate transcript diffusion.

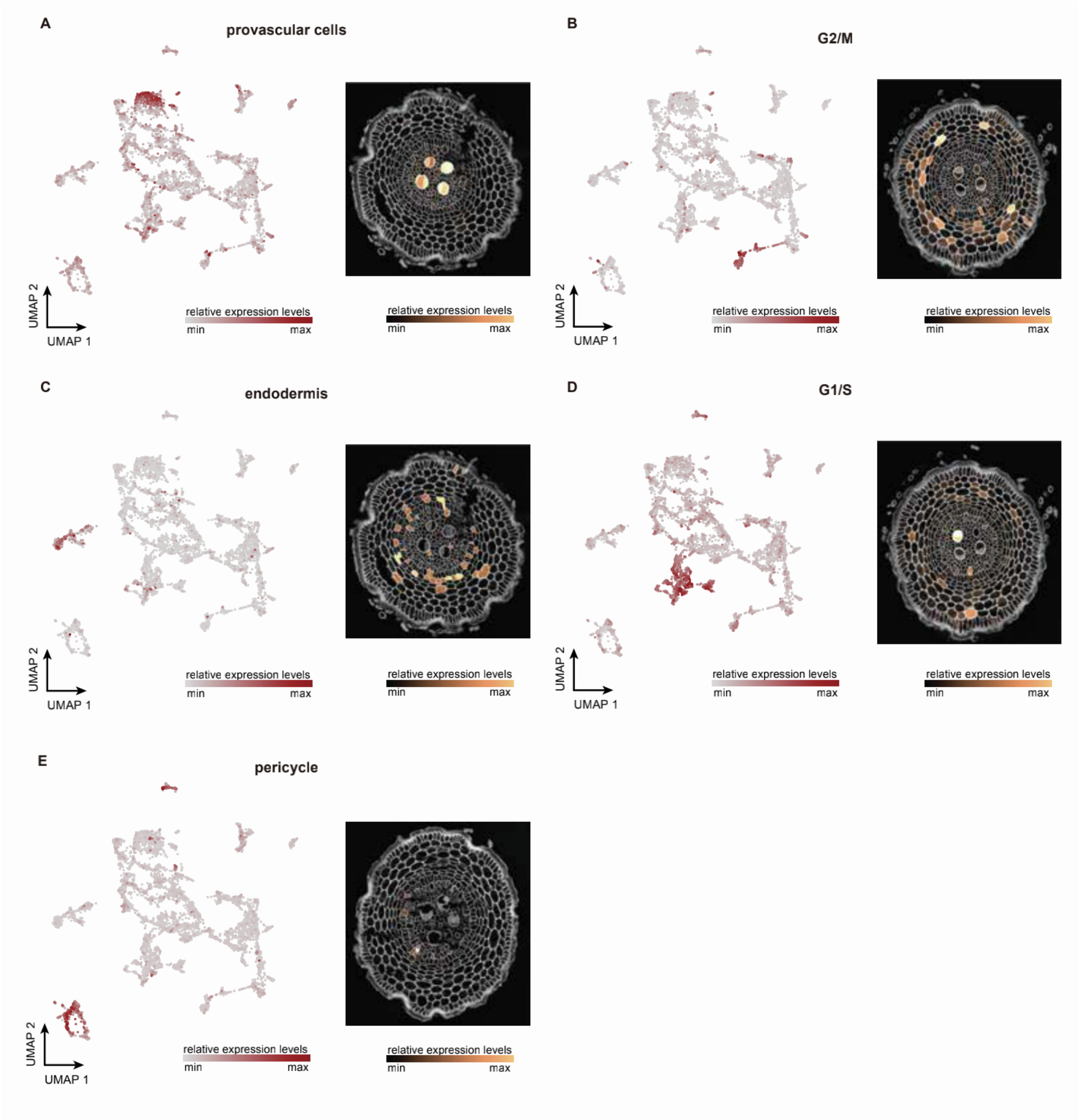

Figure S4. scRNA-seq derived marker gene expression patterns in Stereo-seq root sections. (**A-D**) UMAP feature plot and Stereo-seq visualization of marker genes from provascular cells (A), G2/M (B), endodermis (C), G1/S (D), pericycle (E).

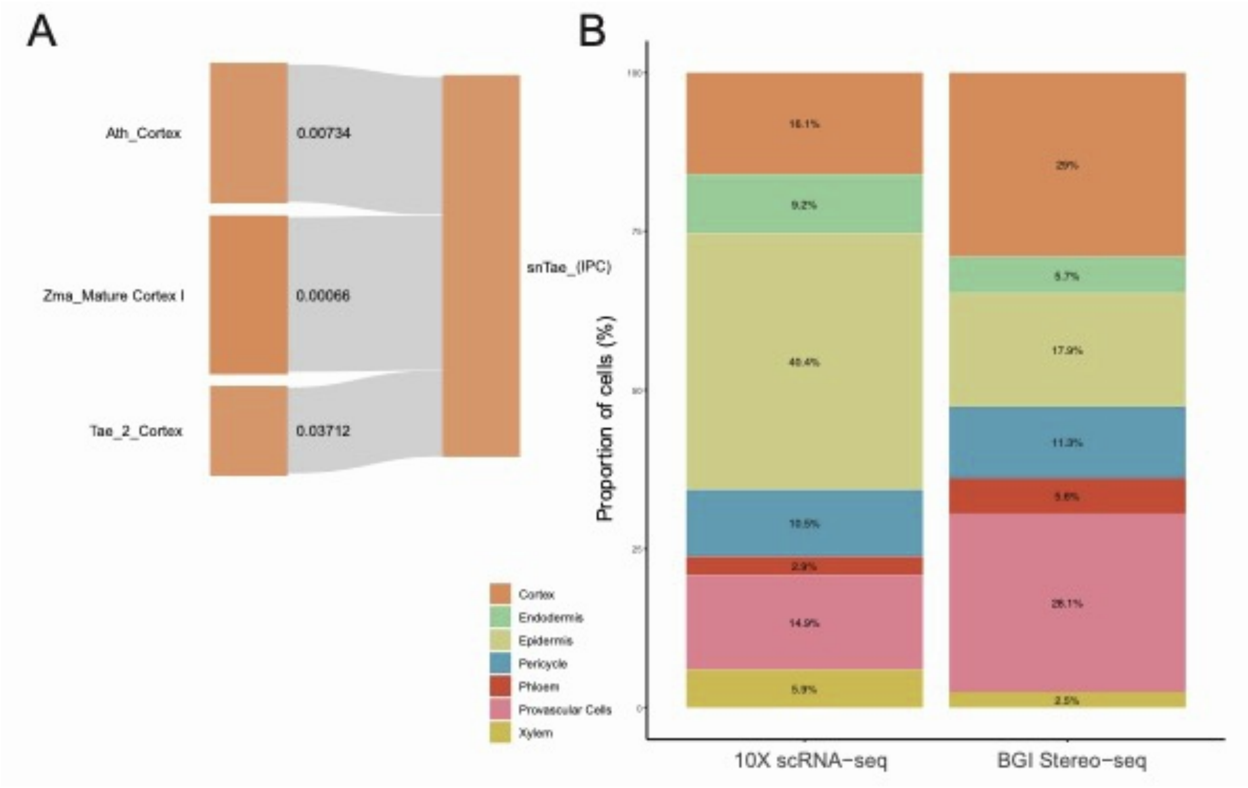

Figure S5. Resolved cell type annotation and cell type composition of single cell (10X genomics) and spatial transcriptomics (STOmics Stereo-seq) dataset. (A) Sankey plot showing resolved annotations transferred from Arabidopsis (*Ath*), maize (*Zma*), wheat (*Tae*) to snTae_Immature Pericycle Cells (IPC) and corresponding q-value. (**B**) Proportion of major cell types in the scRNA-seq and spatial transcriptomics dataset for comparison.

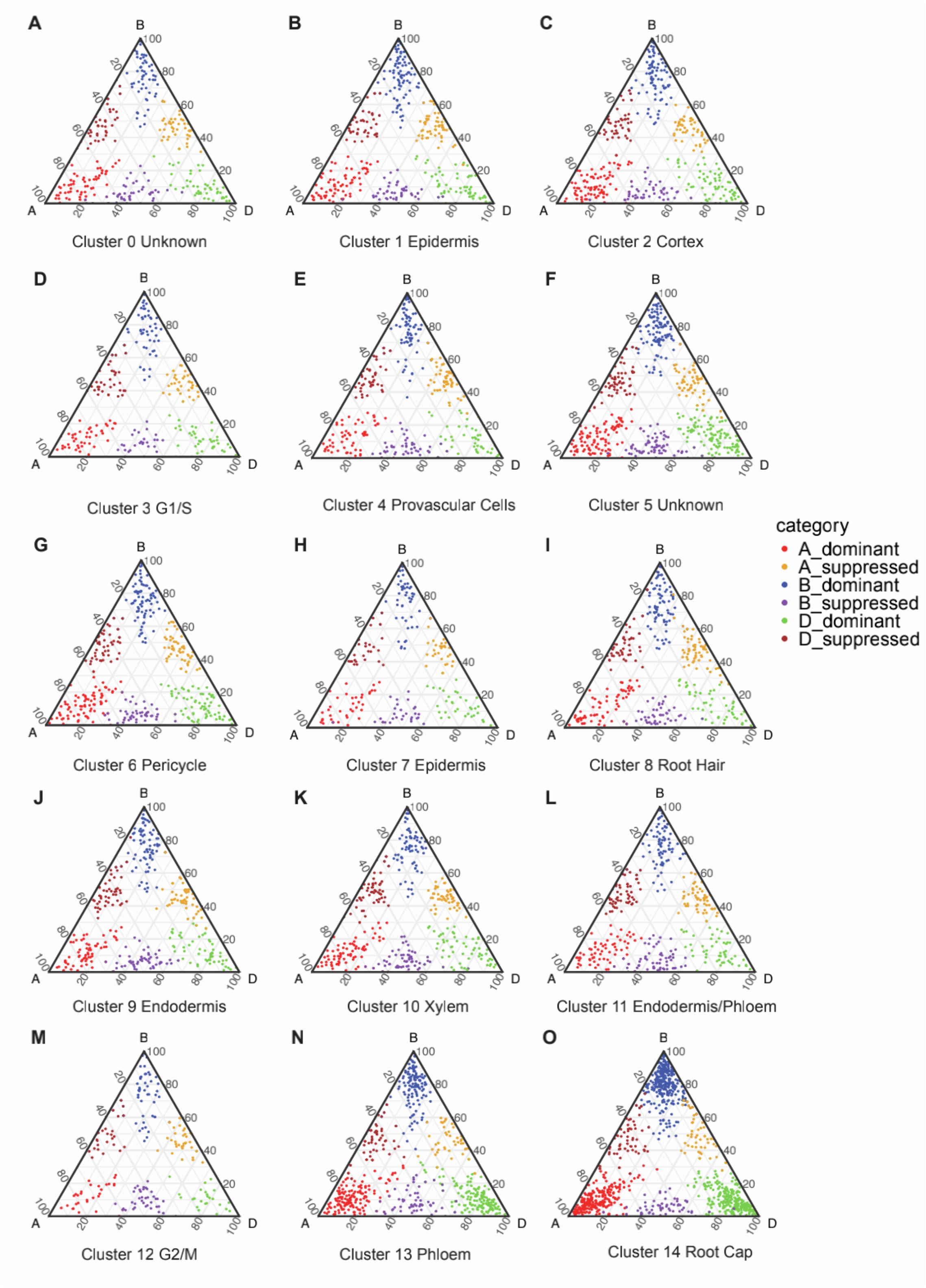

Figure S6. Ternary plots showing genome asymmetry distribution across all clusters.

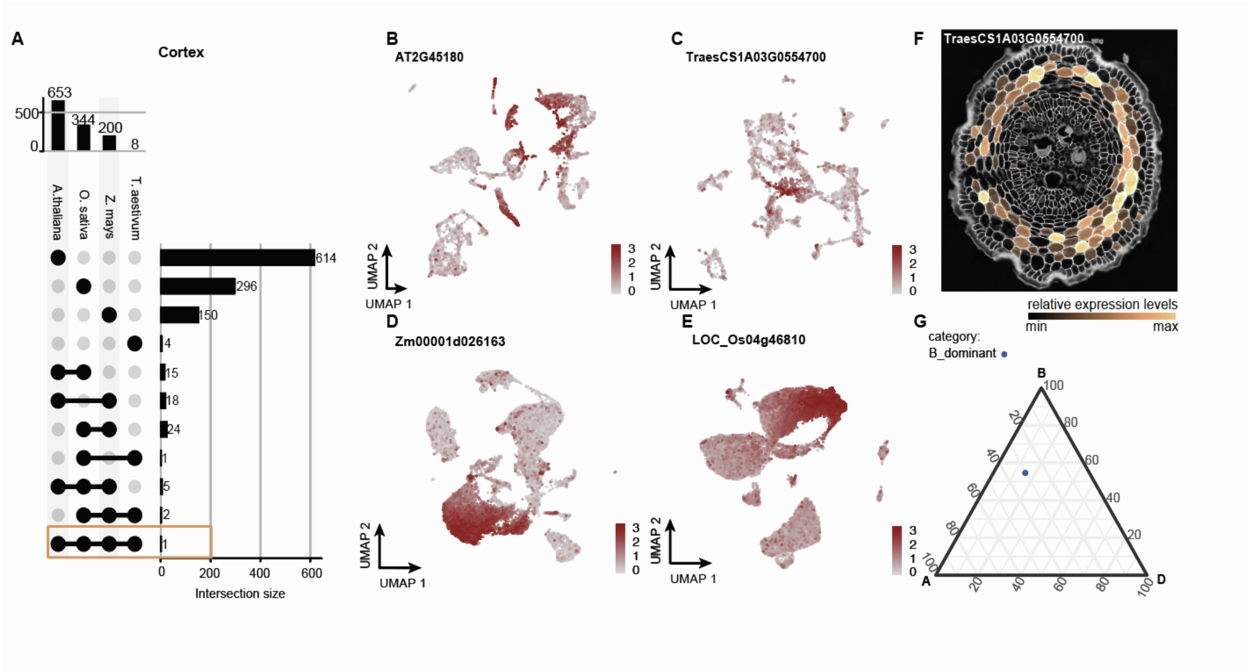

Figure S7. Tissue-specific markers conserved across Arabidopsis, wheat, rice, and maize. (**A**) UpSet plot showing the intersections of cortex markers across Arabidopsis, wheat, rice, and maize. (**B-E**) Feature plots of a cortex specific marker across species. (**F-G**) Spatial expression in Stereo-seq data (F) and ternary plot showing genome asymmetry information (G) of the same cortex specific marker in the wheat root meristem.

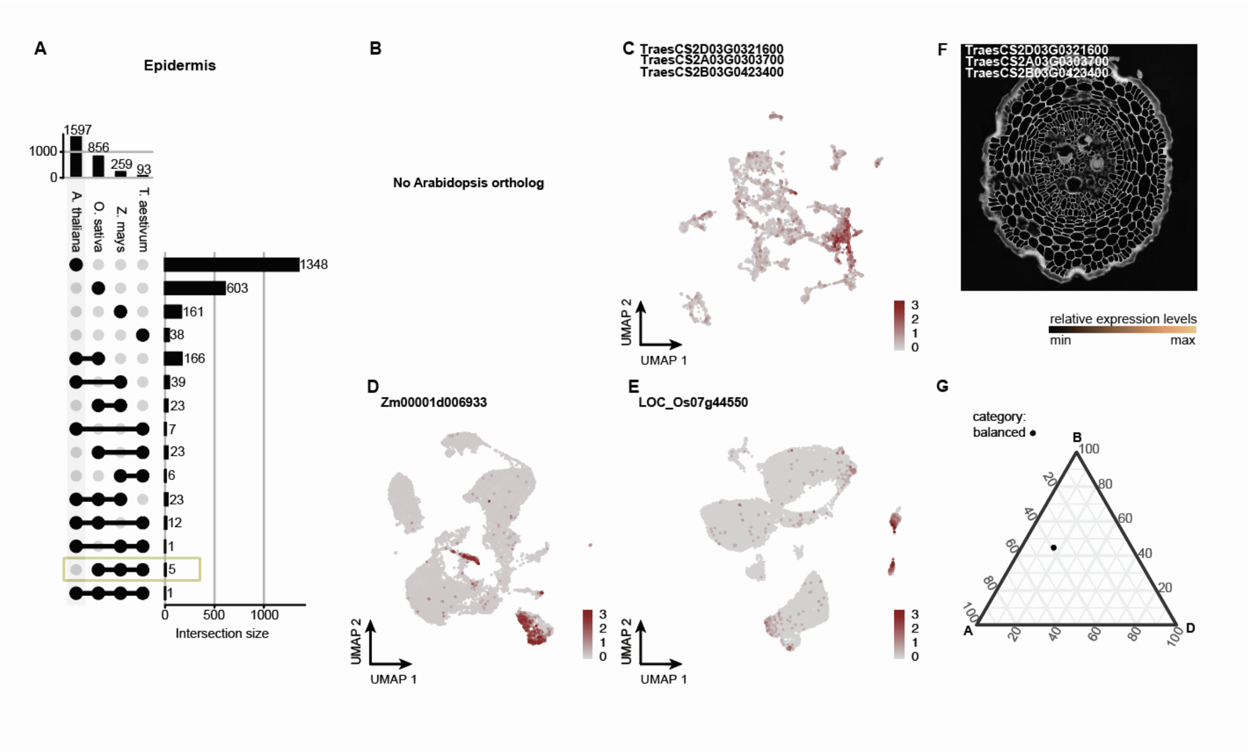

Figure S8. Tissue-specific markers unique to monocot clade (wheat, rice, and maize). (**A**) UpSet plot showing the intersections of epidermis markers across Arabidopsis, wheat, rice, and maize. (**B-E**) Feature plots of an epidermis specific marker across species. (**F-G**) Spatial expression in Stereo-seq data (F) and ternary plot showing genome asymmetry information (G) of the same epidermis specific marker in the wheat root meristem.

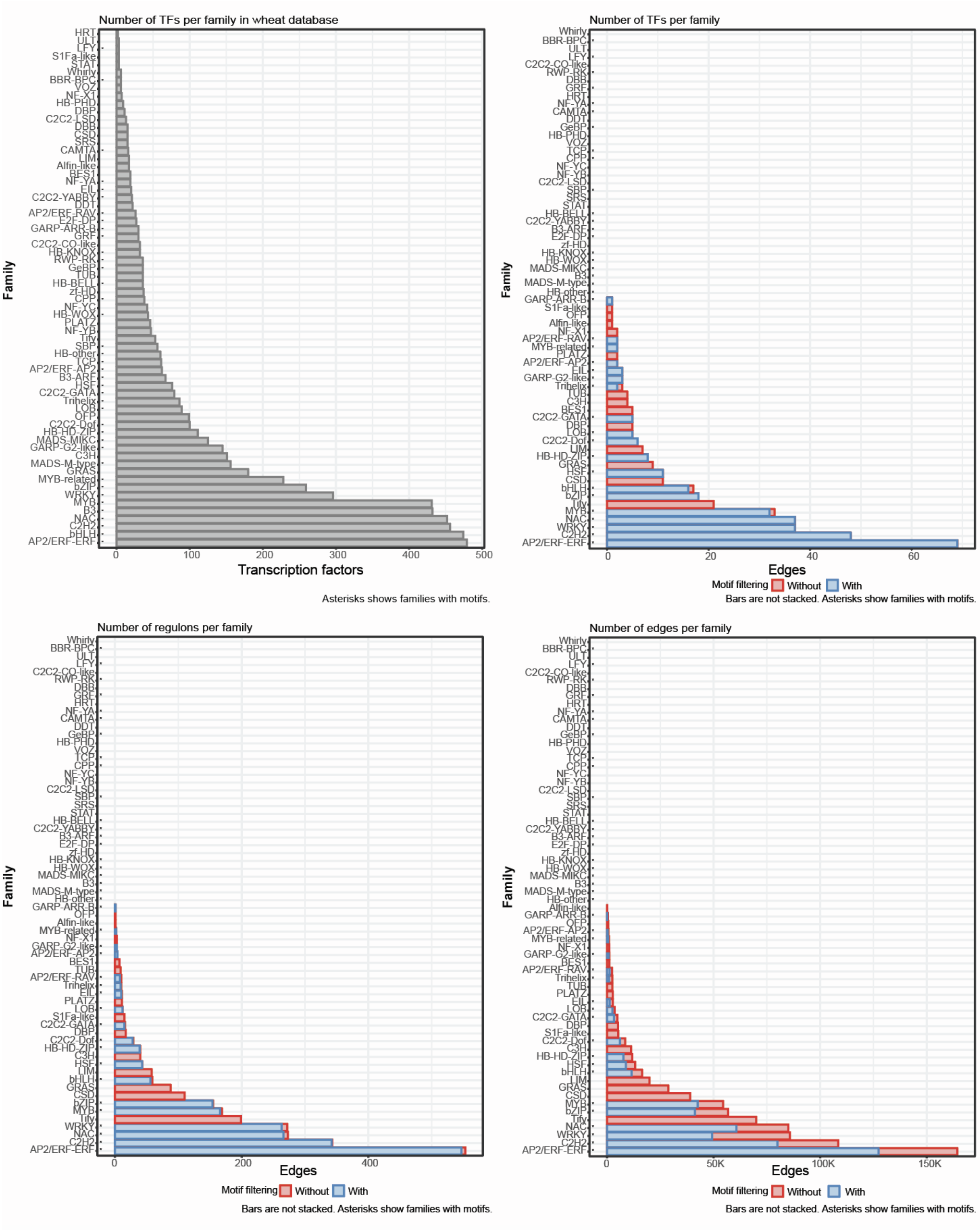

Figure S9. Impact of motif enrichment analysis on MINI-EX results for wheat. (A) Distribution of TFs across TF families within the MINI-EX wheat database. (B-C-D) Comparison of TFs, regulons and edges across TF families in MINI-EX results with or without motif enrichment analysis. Some families are entirely excluded from the GRN as they lack associated motifs.

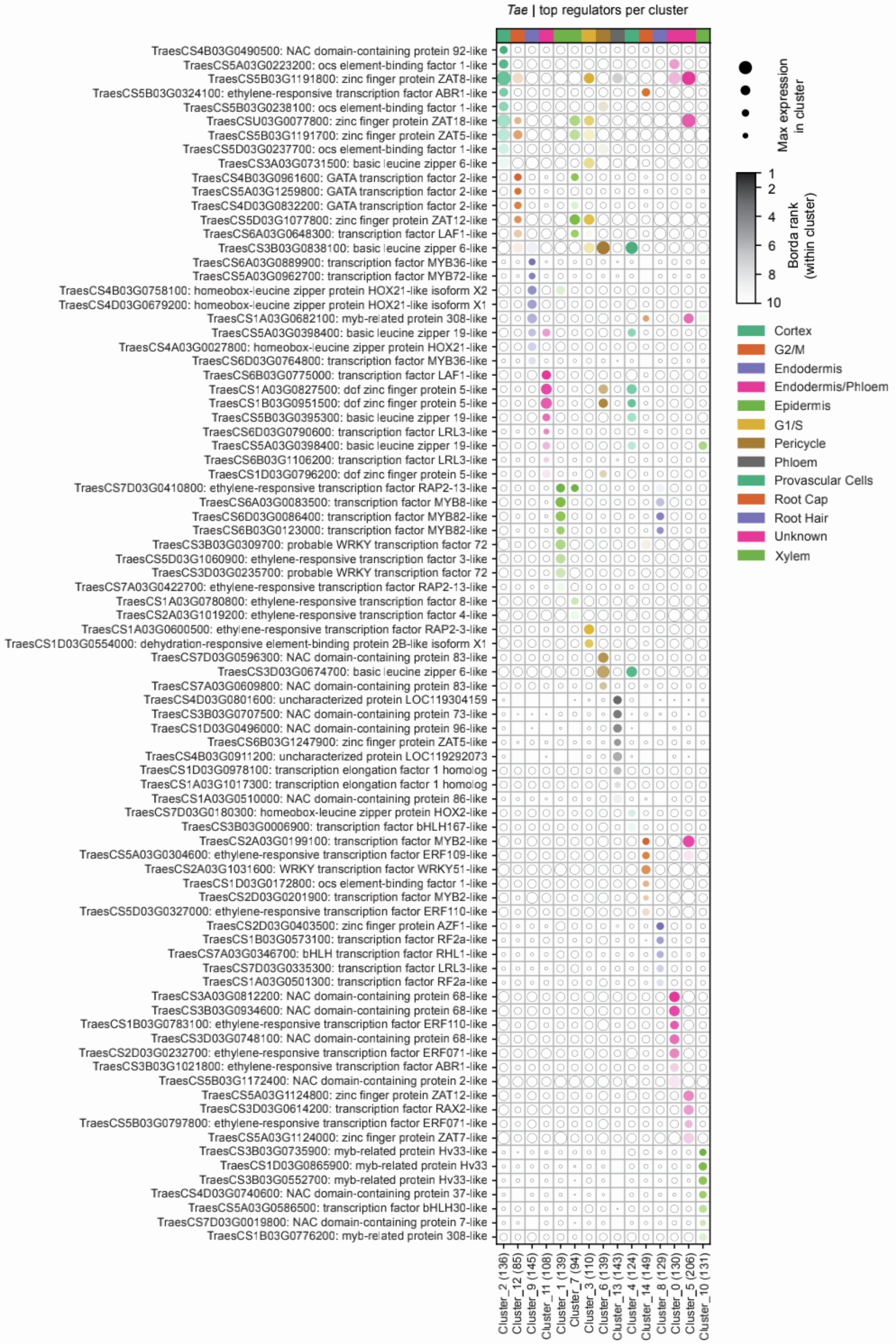

Figure S10. Top regulons for each cluster for wheat (*Tae*).

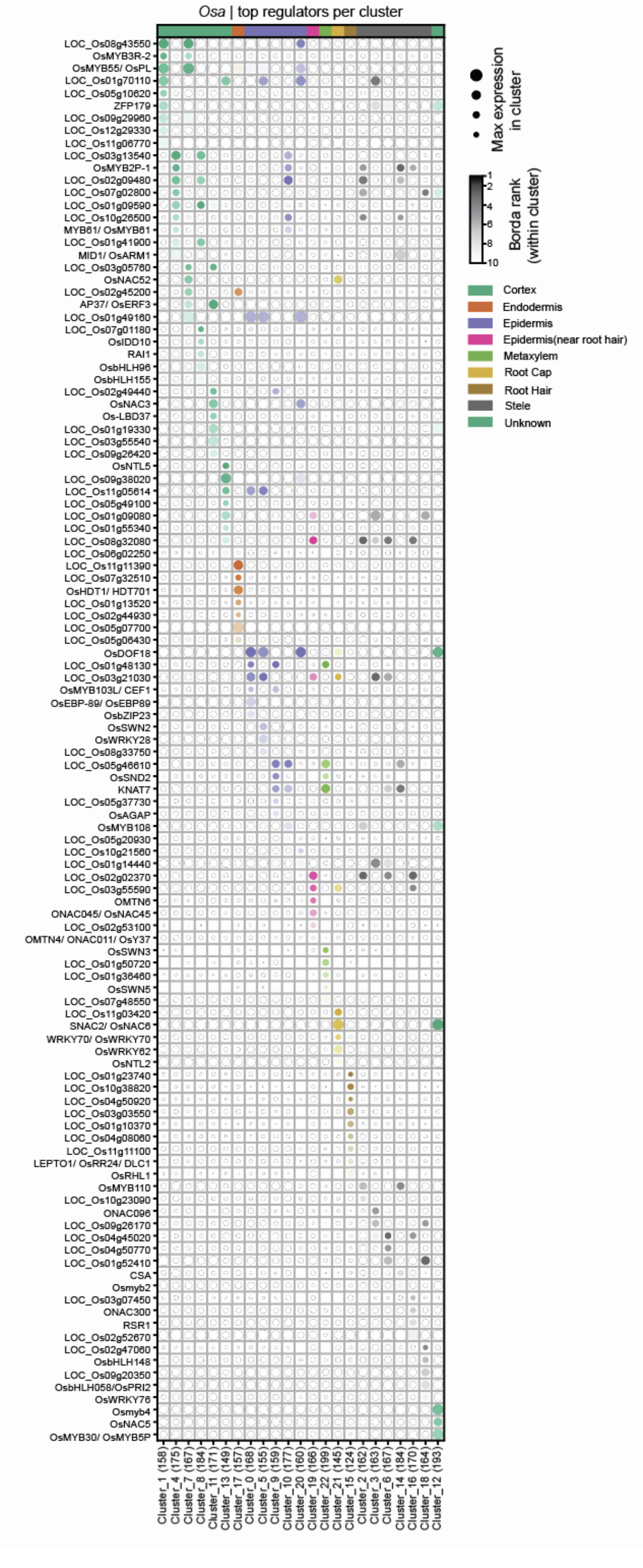

Figure S11. Top regulons for each cluster for rice (*Osa*).

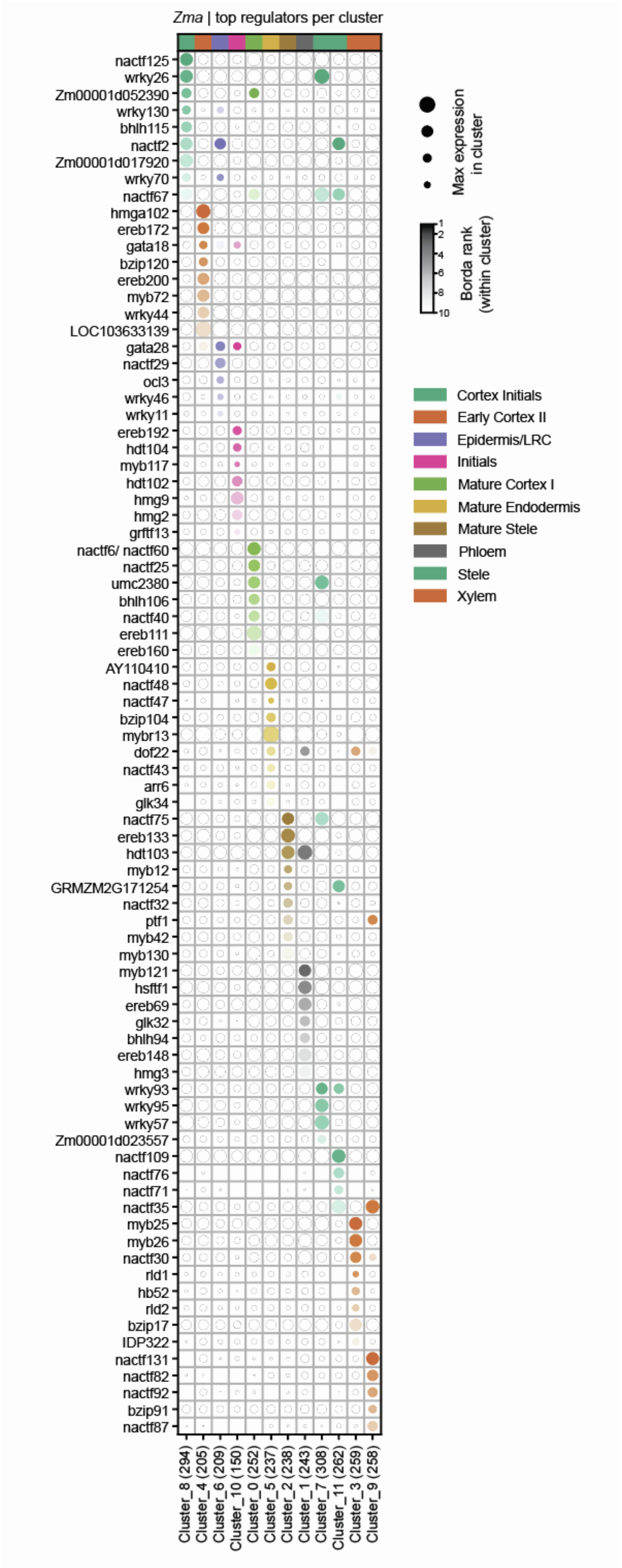

Figure S12. Top regulons for each cluster for maize (*Zma*).

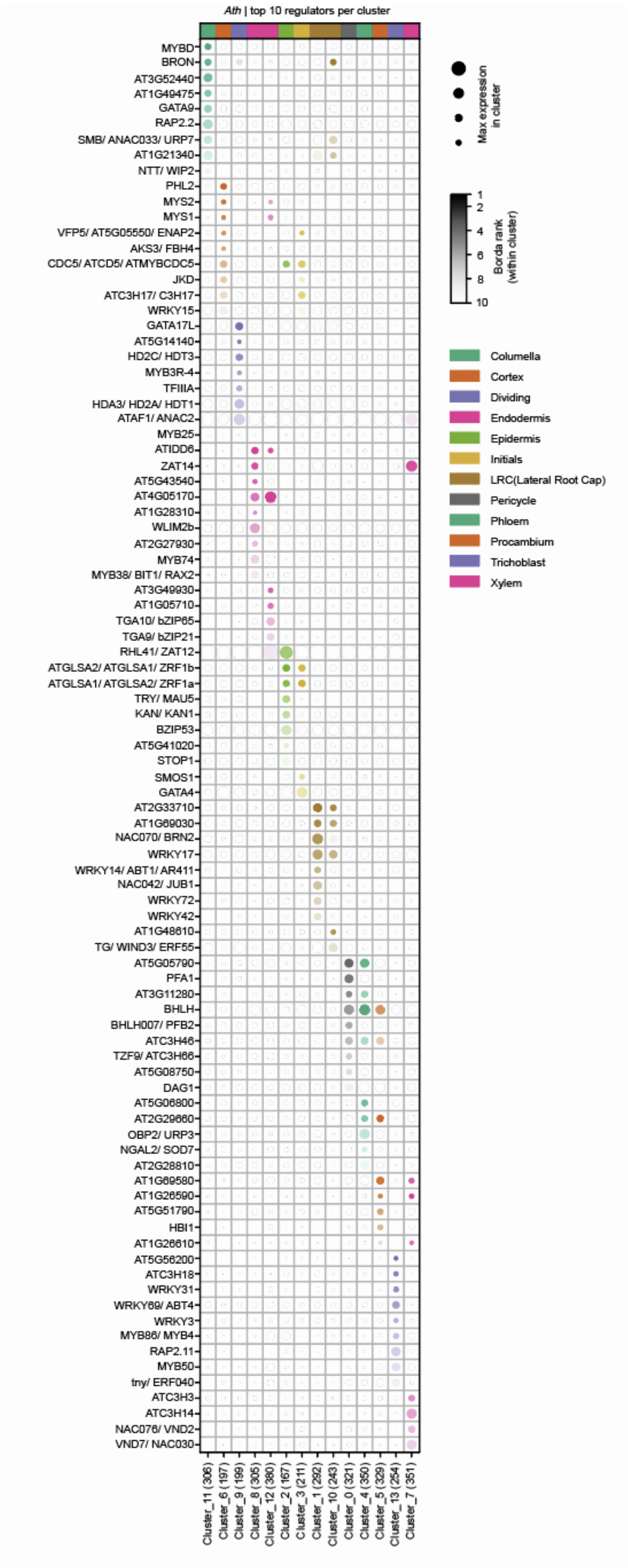

Figure S13. Top regulons for each cluster for Arabidopsis (*Ath*).
